# Notes on the subfamily Pleganophorinae, with the description of a new species of the genus *Trochoideus* Westwood, 1833 (Coleoptera, Endomychidae) from China

**DOI:** 10.3897/zookeys.1290.183211

**Published:** 2026-08-26

**Authors:** Pan-Pan Li, Ming Bai, Ling-Xiao Chang

**Affiliations:** 1 State Key Laboratory of Animal Biodiversity Conservation and Integrated Pest Management, Institute of Zoology, Chinese Academy of Sciences, Beijing 100101, China State Key Laboratory of Animal Biodiversity Conservation and Integrated Pest Management, Institute of Zoology, Chinese Academy of Sciences Beijing China; 2 Guangxi Laboratory of Forestry, Guangxi, Nanning 5300024, China Guangxi Forestry Research Institute Nanning China; 3 Guangxi Forestry Research Institute, Guangxi, Nanning 5300024, China Guangxi Laboratory of Forestry Nanning China; 4 Department of Life Sciences, Natural History Museum of China, Beijing 100050, China Department of Life Sciences, Natural History Museum of China Beijing China

**Keywords:** Biodiversity, Coccinelloidea, Coleoptera, fungus beetles, identification key, new species, Oriental Region, taxonomy

## Abstract

The subfamily Pleganophorinae (Coleoptera, Endomychidae), an unusual group distinguished by its distinctive antennal morphology, is reported for the first time in mainland China with the description of *Trochoideus sinensis***sp. nov**. from Guangdong and Jiangxi and the first records of *T. desjardinsi* Guérin-Méneville, 1838 and *T. tonkineus* Strohecker, 1980 from Hainan. Additionally, the male of *Dadocerus nitidus* Arrow, 1920 is discovered for the first time, and the sex assignment in the original description is corrected. Morphological diagnoses are augmented by comparative analyses of antennal dimorphism and mitochondrial cytochrome *c* oxidase I (COI) gene barcoding data. A key to the Chinese species of *Trochoideus* is provided.

## Introduction

**Figure 1.**
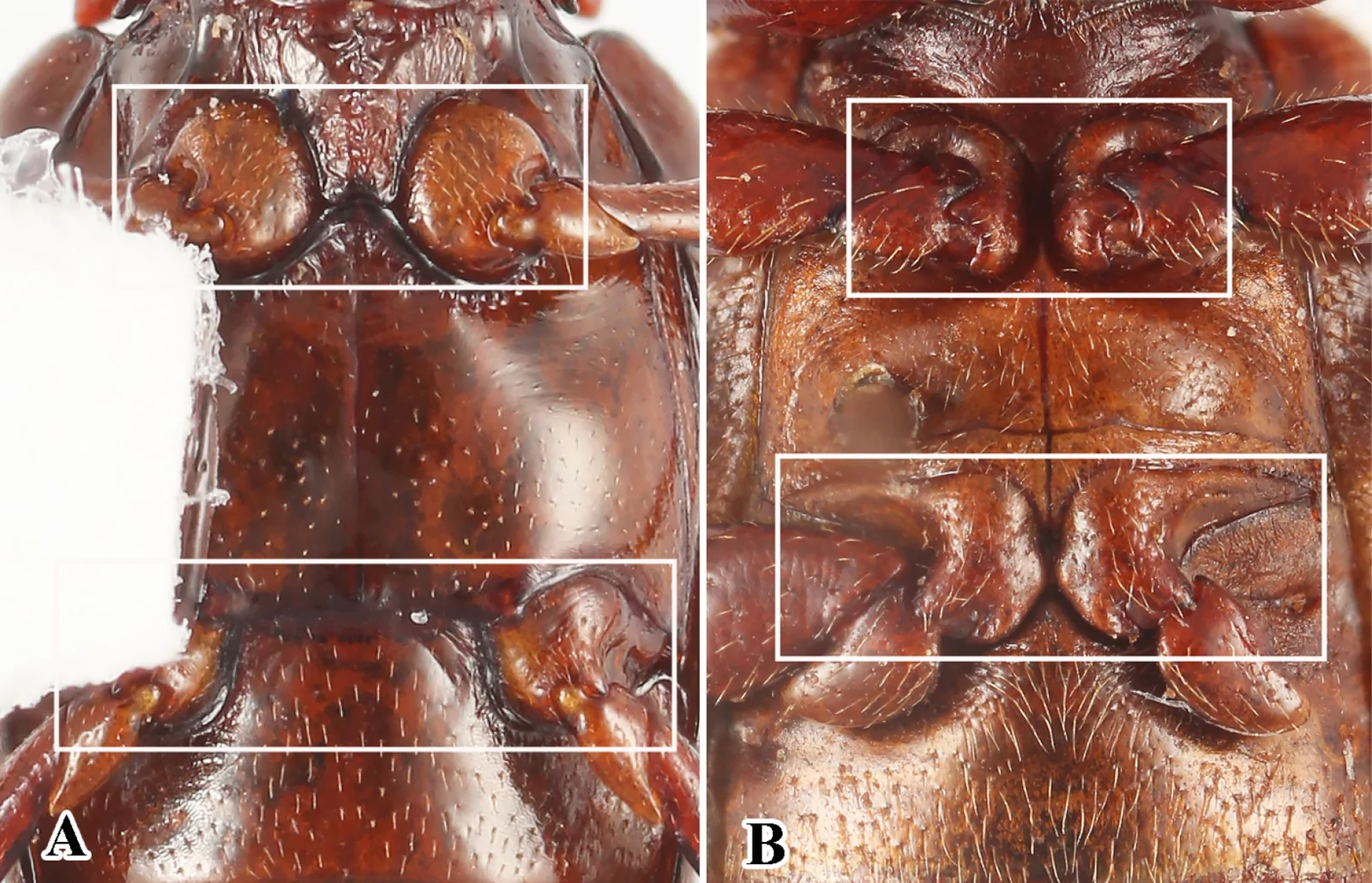
Mesocoxae and metacoxae of Pleganophorinae and Paussinae. **A**. Pleganophorinae (*Dadocerus nitidus*); **B**. Paussinae (*Platyrhopalus paussoides*).

Pleganophorinae Jacquelin du Val, 1858, is a small subfamily within Endomychidae, comprising only three genera and 21 species worldwide (Shockley et al. 2009a). This subfamily is distinguished from other endomychids primarily by its highly modified and distinctive antennal morphology, which superficially resembles that of some Paussinae (Carabidae) (Bell and Bell 1978). However, Pleganophorinae can be clearly separated from Paussinae by the mesoventrite with intercoxal process trapezoidal in shape, and moderately widely separating mesocoxae (vs. intercoxal process of mesoventrite very narrow, mesocoxae almost touching); metaventrite without intercoxal process posteriorly, and metacoxae widely separating (vs. very narrow, metacoxae almost touching) (Fig. 1). The pronounced antennal enlargement and reduction in number of antennomeres observed in this group may represent an adaptation to a symbiotic lifestyle associated with social insects such as ants and termites, as discussed in Shockley et al. (2009b).

The subfamily is characterized and diagnosed by the following combination of morphological traits: antennae 4- or 5-segmented in male, some species have 7 antennomeres in females, with the terminal one or two antennomeres conspicuously enlarged, especially in males; mandibles produced and finely cleft apically; labium with nearly quadrate terminal palpomere; maxillary palps subcylindrical; prosternal process narrowly separates the procoxae; mesoventral process also narrow; and tarsi 4-segmented, stout in *Pleganophorus* but slender in the other genera (Strohecker 1953).

Within the subfamily Pleganophorinae, *Dadocerus* Arrow, 1920 is restricted to Borneo, while *Pleganophorus* Hampe, 1855 occurs in Central and Eastern Europe. The largest genus, *Trochoideus* Westwood, 1833, is widely distributed across tropical regions worldwide and represents the only genus of Pleganophorinae currently recorded from China (Shockley et al. 2009a; Berrazueta et al. 2024).

Westwood (1833) established the genus *Trochoideus* with its type species based on a fossil specimen preserved in gum copal. This specimen had been previously described and named *Paussus cruciatus* (Carabidae, Paussinae) by Dalman (1825). Prior to this study, *Trochoideus* comprised nineteen species, including one species recorded from China (Taiwan): *T. desjardinsi* Guérin-Méneville, 1838 (Shockley et al. 2009a; Berrazueta et al. 2024; Shûhei Nomura et al. 2025).

In the present study, *Trochoideus sinensis* sp. nov. is described as a new species from China, and *Trochoideus tonkineus* is newly recorded from China, with the female of the latter described for the first time. These findings bring the global species count of Pleganophorinae to twenty-two, providing a more robust basis for future phylogenetic and systematic studies within the subfamily.

## Material and methods

Type specimens of the new species described here are deposited in the following institutions and private collections:

**CBWX** Collection of Wen-Xuan Bi, Shanghai, China;

**IZCAS** Institute of Zoology, Chinese Academy of Sciences, Beijing, China;

**NHMC** National Natural History Museum of China, Beijing, China.

The examined specimens of known species are deposited in the following institutions and private collections:

**CCLX** Collection of Ling-Xiao Chang, Beijing, China.

**MHBU** Hebei University Museum, Hebei, China.

**MZPW** Polish Academy of Science, Museum and Institute of Zoology, Warszawa [=Warsaw], Poland.

**NHMC** National Natural History Museum of China, Beijing, China.

**NHMUK** Natural History Museum, London, United Kingdom.

**NSMT** National Science Museum (Natural History), Tokyo, Japan.

The specimens were examined, dissected, and measured using an Olympus SZX10 dissecting microscope. Measurements were standardized as follows: body length (BL) = length between the apex of the clypeus and the elytral apex along the mid-line; pronotum length (PL) = length of the pronotum along the mid-line; pronotum width (PW) = maximum width of the pronotum; elytra length (EL) = length from anterior margin to apices of the elytra; elytra width (EW) = widest part of both elytra combined. Morphological terminology follows Tomaszewska (2005) and Yoshitomi (2020).

Habitus photographs were taken using a Canon EOS 5D Mark III camera with a Canon MP-E 65 mm f/2.8 1-5X Macro Lens, and a Canon MT-24EX Macro Twin Lite Flash was used as a light source. Photographs of the antenna, male genitalia and aedeagi were taken with a Canon EOS 5D Mark III camera fitted with a Mitutoyo M Plan Apo 10X microscope objective combined with a WeMacro M26-to-M42 tube lens, and two Canon 430EX III-RT Macro Twin Lite flashes were used as light sources. The initial images were then stacked in Helicon Focus v. 8.1.1 and refined in Adobe Photoshop CC 2023.

A specimen of *Trochoideus sinensis* sp. nov. was preserved in anhydrous ethanol, and transferred into a 1.5 ml microcentrifuge tube and air-dried for several hours until the ethanol had completely evaporated. Genomic DNA was extracted using the DNeasy® Blood and Tissue Kit (QIAGEN, Germany) following the manufacturer’s instructions. A fragment of the mitochondrial COI gene was amplified by PCR in a 25 μl reaction mixture containing 12.5 μl of 2×Taq PCR Mix (TIANGEN, China), 1 μl of each primer (10 μM; LCO1490: 5'-GGTCAACAAATCATAAAGATATTGG-3', HCO2198: 5'-TAAACTTCAGGGTGACCAAAAAATCA-3'), 2 μl of DNA template, and 8.5 μl of ddH_2_O. The PCR conditions were 94 °C for 3 min, followed by 35 cycles of 94 °C for 45 s, 48 °C for 45 s, and 72 °C for 45 s; and the final extension at 72 °C for 6 min. Forward and reverse Sanger sequencing were performed by the Tsingke Company (Beijing, China). The resulting sequence was deposited in the GenBank database under the accession number PX696962.

## Taxonomy

### Subfamily Pleganophorinae

Taxon classificationAnimaliaEndomychidae

F87141F0-51C1-5E96-BC5D-EE884B09B15B

Pleganophorinae Jacquelin du Val, 1858: 186.
Endomychidae adsciti Gerstaecker, 1858: 377 [*sed. dub*.].
Paussoideidae Gorham, 1873: 29.
Trochoideites Chapuis, 1876: 146 [*sed. dub*.].
Pleganophorini Reitter, 1881: 926.
Trochoideinae Ganglbauer, 1899: 926.

### Genus Dadocerus

Taxon classificationAnimaliaColeopteraEndomychidae

Arrow, 1920

6C774072-5788-5181-8B1C-48ECA11DA8A7

Dadocerus Arrow, 1920: 76.

#### Description (modified from Arrow 1920).

Body 4.5–5.0 mm long; bright chestnut-brown; weakly convex. Dorsal smooth and glossy; densely and finely punctate. Antenna 4- segmented, almost as long as head and thorax together; terminal antennomeres greatly enlarged, especially in male. Mandible strongly sharp and without subapical teeth. Maxilla with terminal palpomere longer than 2 and 3, strongly tapering and oblique truncate apically. Labium with palpi narrowly separated at base, terminal palpomere thick and large, nearly bowl-shaped. Pronotum transverse, broad anteriorly, strongly narrowed posteriorly, with a longitudinal median groove; the groove bearing two fine lines; all angles lobed, not acute; elytra strongly elongate, posteriorly tapering, separately rounded at apex; humeri prominent and ridged, the ridge extending posteriorly to about basal 1/3 of elytron; second shorter inner ridge present, along with a deep groove next to suture. Tarsal formula 4-4-4 in both sexes. Abdomen with six freely articulated ventrites.

#### Diagnosis.

The *Dadocerus* resembles two other genera of Pleganophorinae in appearance. However, the pronotum with a distinctly median longitudinal sulcus can separate it from *Pleganophorus* (Fig. 8) and *Trochoideus* (Fig. 9) (Strohecker 1953).

### Dadocerus nitidus

Taxon classificationAnimaliaColeopteraEndomychidae

Arrow, 1920

6594CF6B-4B2F-5646-AE38-FDCD7DB2FF2E

**Figure 2.**
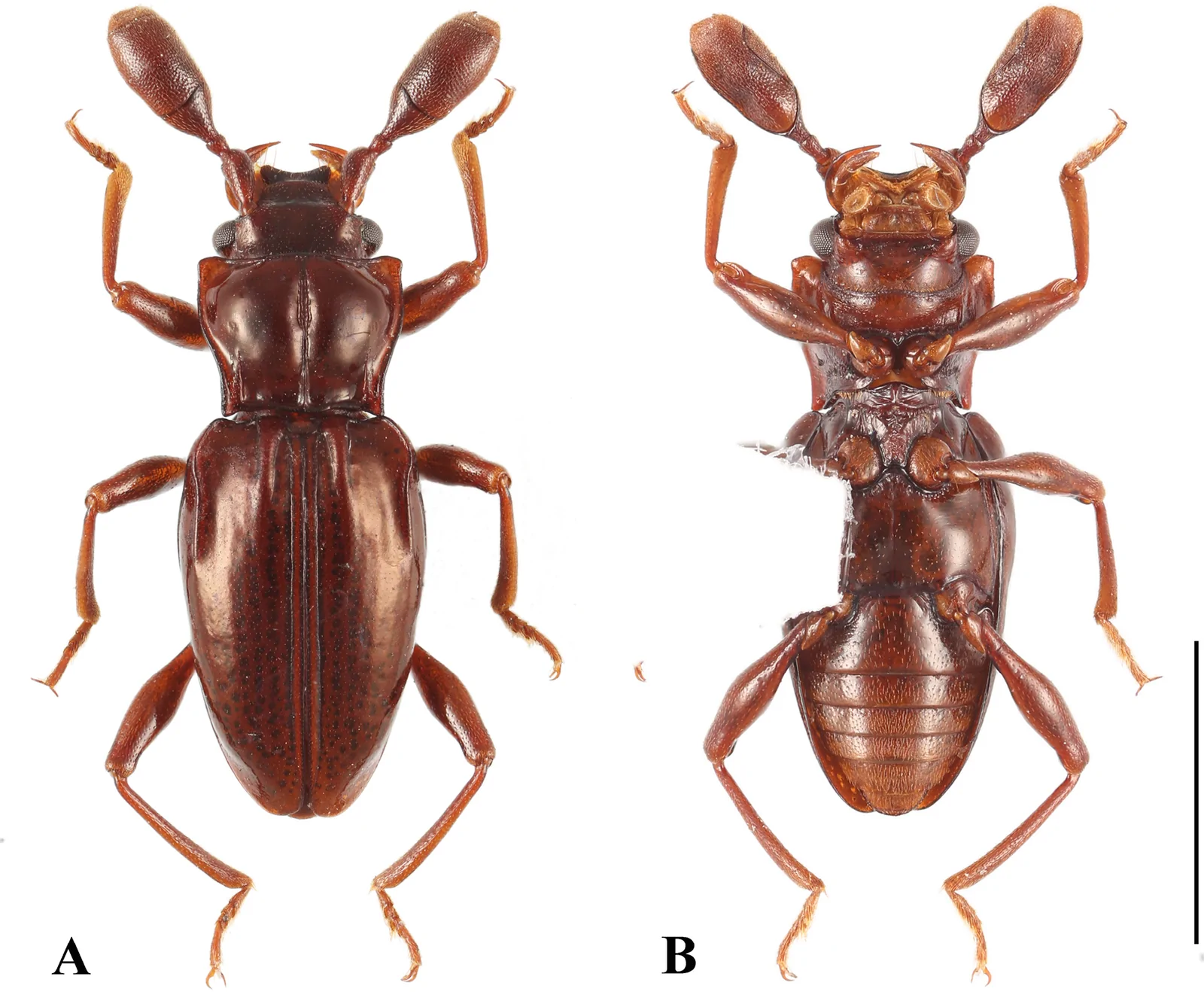
Habitus of *Dadocerus nitidus*, male. **A**. Dorsal view; **B**. Ventral view. Scale bars: 1.0 mm.

**Figure 3.**
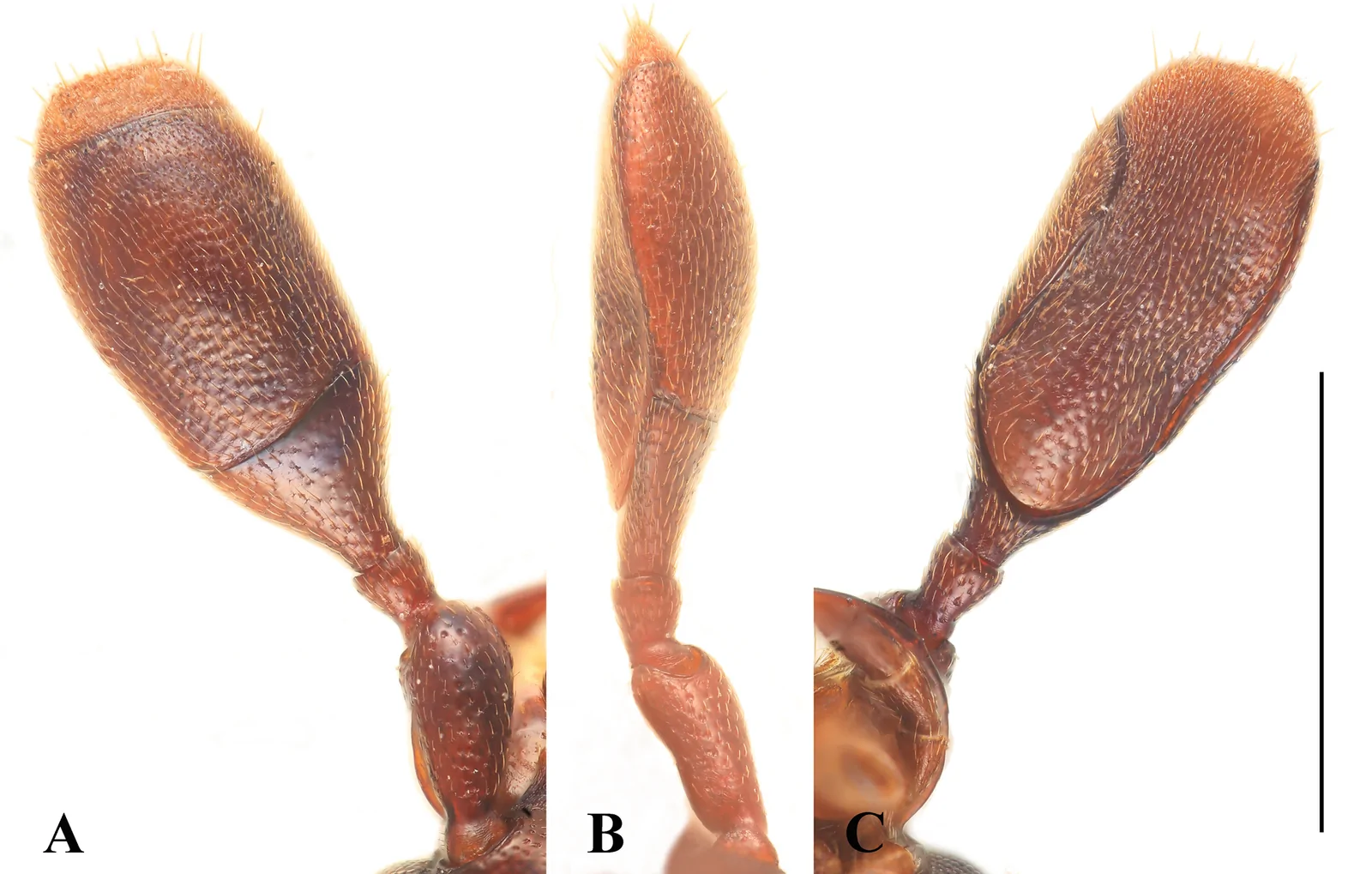
Left antenna of *Dadocerus nitidus* (male). **A**. Dorsal view; **B**. Lateral view; **C**. Ventral view. Scale bar: 1.0 mm.

**Figure 4.**
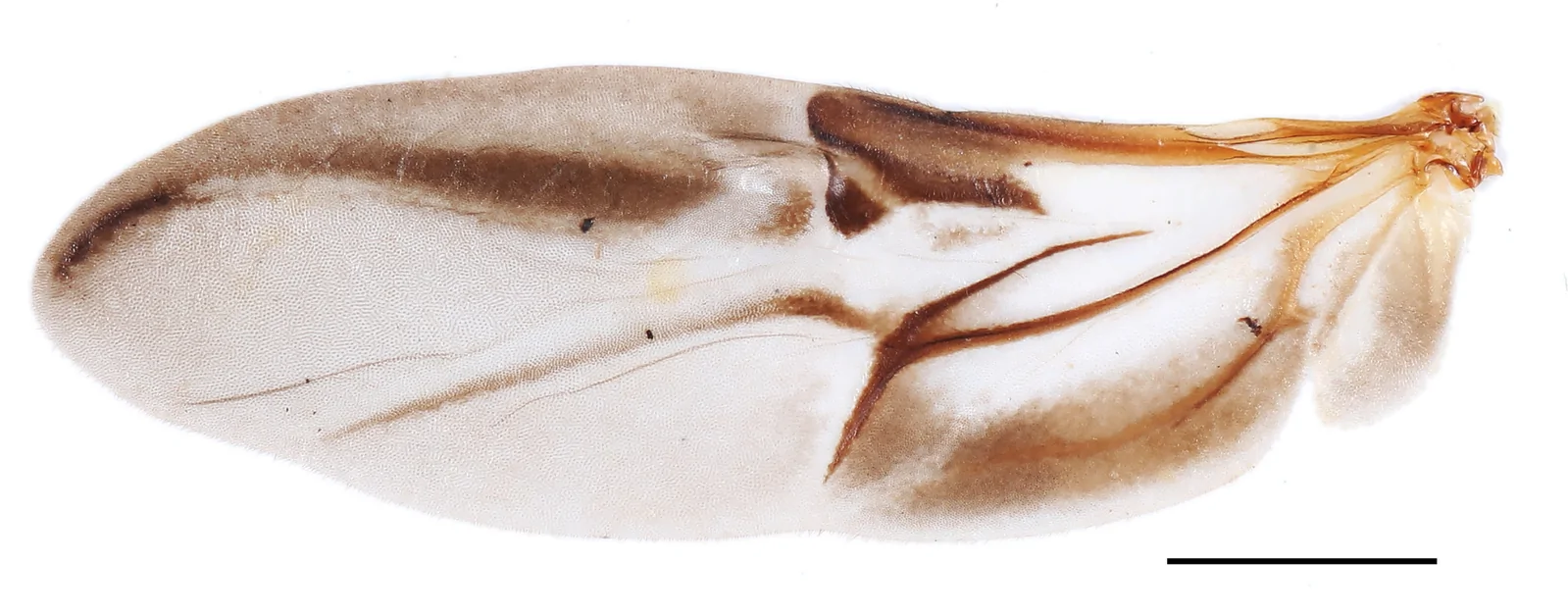
Left hind wing of *Dadocerus nitidus*. Scale bar: 1.0 mm.

**Figure 5.**
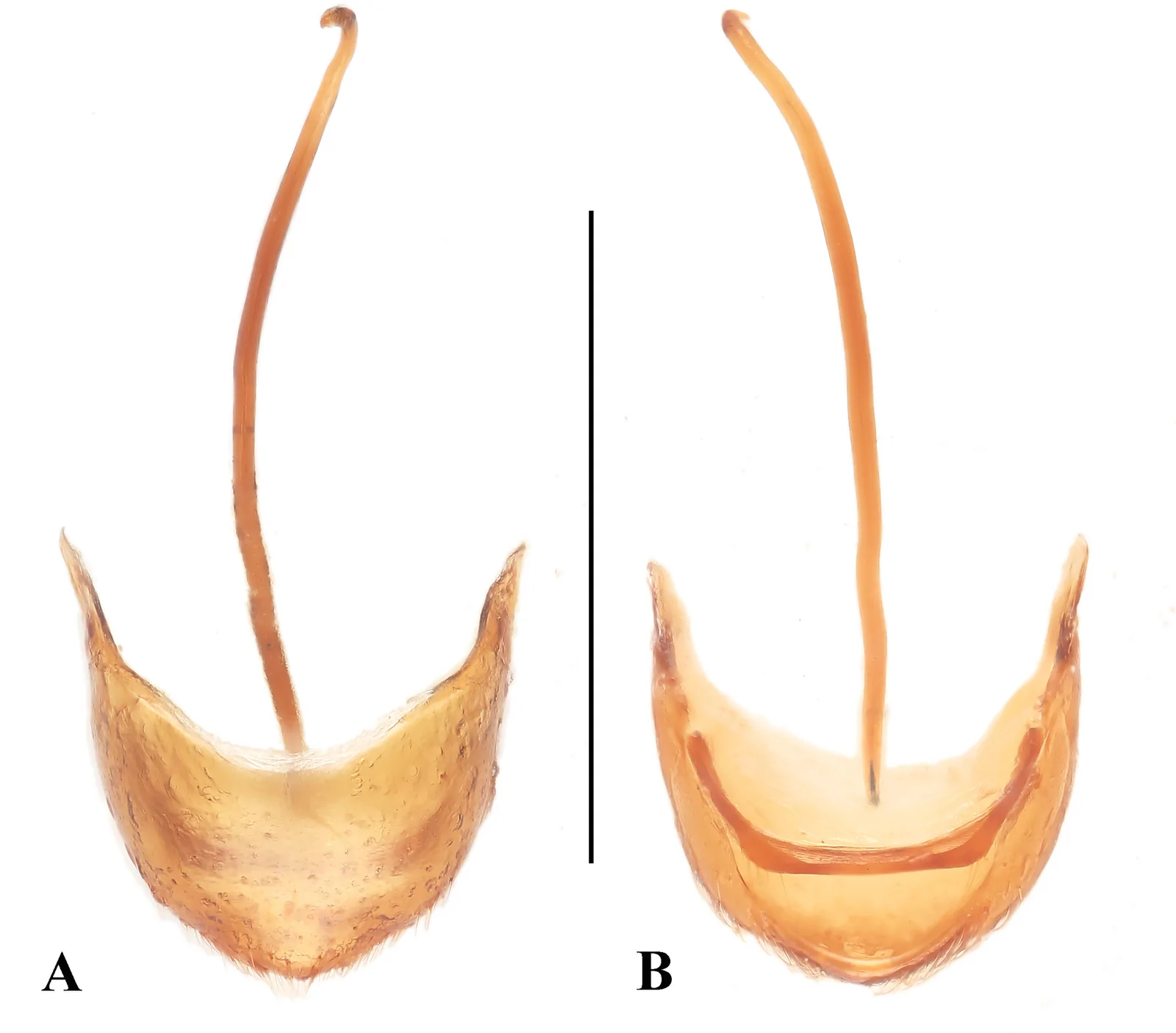
Male genital segment of *Dadocerus nitidus*. **A**. Dorsal view; **B**. Ventral view. Scale bar: 1.0 mm.

**Figure 6.**
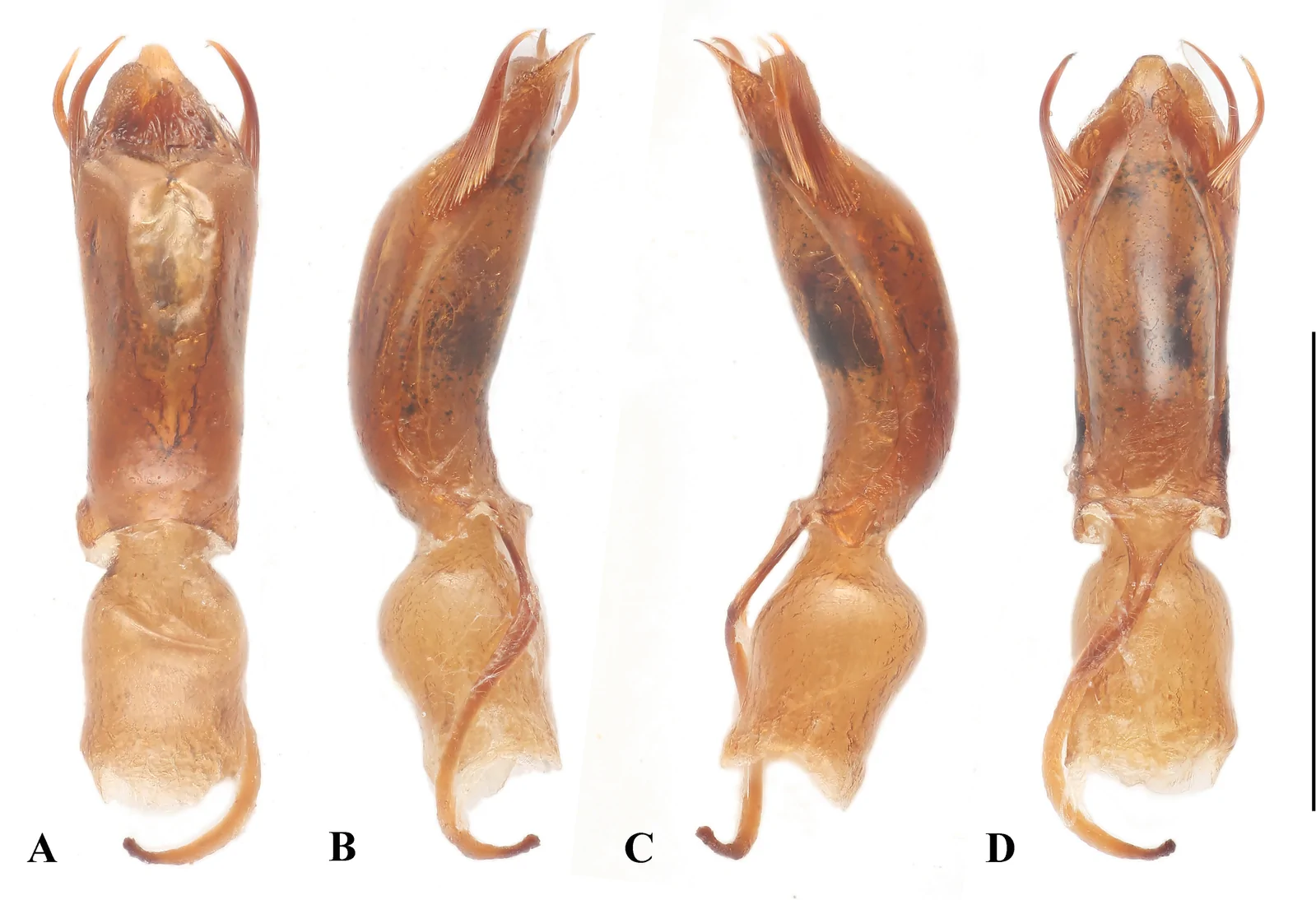
Aedeagus of *Dadocerus nitidus*. **A**. Dorsal view; **B**. Left lateral view; **C**. Right lateral view; **D**. Ventral view. Scale bar: 1.0 mm.

**Figure 7.**
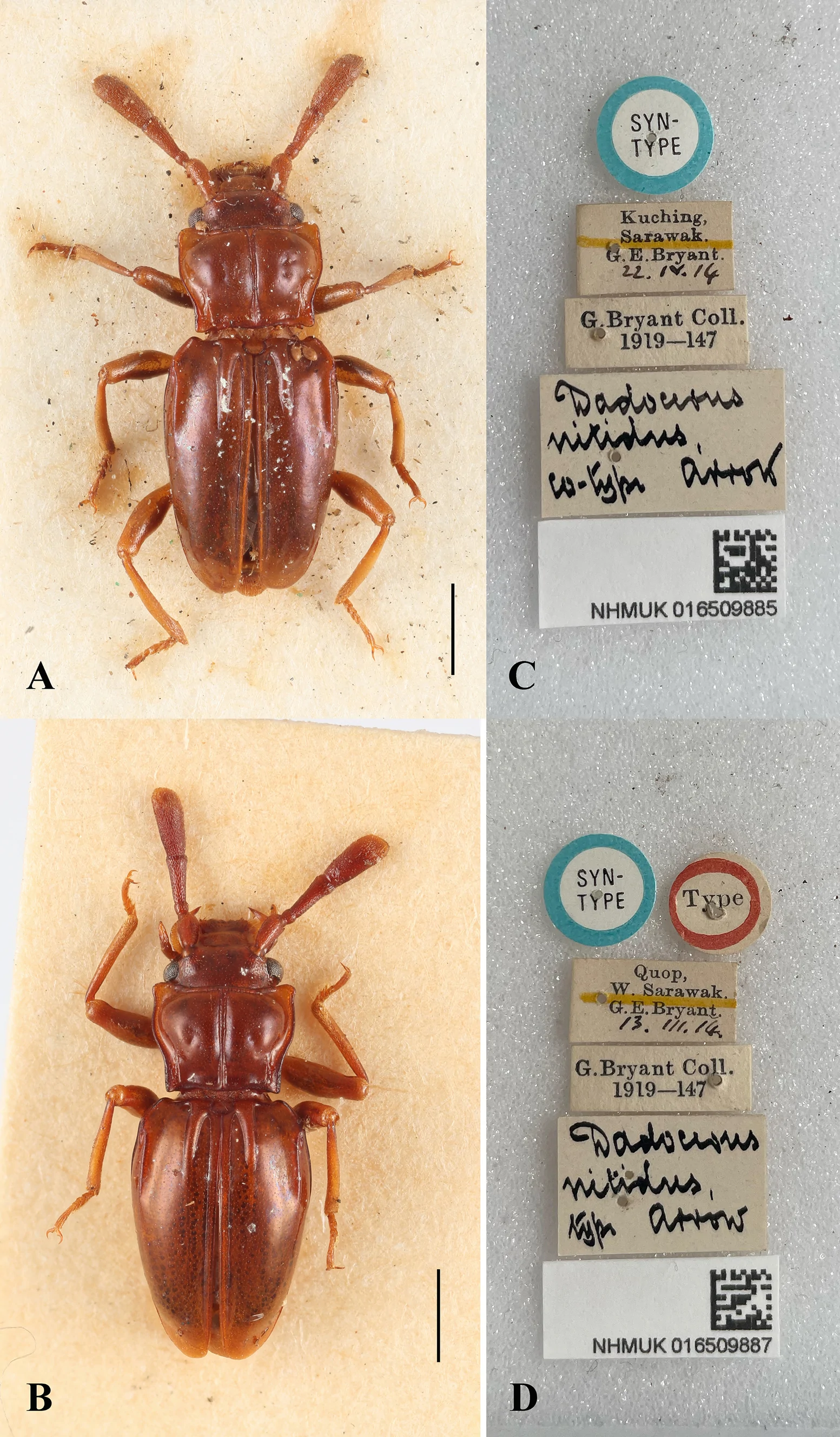
Habitus of *Dadocerus nitidus*, female. **A, B**. Dorsal view; **C, D**. Labels. Scale bar: 1.0 mm.

Figs 2, 3, 4, 5, 6, 7

Dadocerus nitidus Arrow, 1920a: 76.

#### Type material examined.

***Lectotype*** (here designated): Malaysia • ♀; glued on a card with five labels as follows: “SYN-TYPE” “Kuching, Sarawak. G. E. Bryant. 22. IV. 14” “G. Bryant Coll. 1919–147” “*Dadocerus nitidus* co-type arrow” “NHMUK 016509885”; (NHMUK). ***Paralectotype*** (here designated): Malaysia • 1♀; glued on a card with six labels as follows: “SYN-TYPE” “Type” “Quop, W. Sarawak. G. E. Bryant. 13. III. 14.” “G. Bryant Coll. 1919–147” “*Dadocerus nitidus* type arrow” “NHMUK 016509887”; (NHMUK).

#### Additional material examined.

Malaysia – Sabah • 5♂♂4♀♀; Papar, 16 Miles; 2026.III.25; Tao-Qi Wang leg.; (CCLX); Sabah • 4♀♀; Mt. Kinabalu; 2024.IV.20; Tao-Qi Wang leg.; (CCLX).

#### Description of male.

***Body*** (Fig. 2) long and oval, 2.6 times as long as wide; weakly convex; smooth, rather shiny. Dorsal and ventral surfaces bright chestnut-brown, eyes black.

***Head***. Antenna (Fig. 3) 4-segmented, scapus large, clavate, with inner margin arcuate, about 2.0 times longer than wide; pedicel longer than wide, base partially obscured covered by scape, concave medially; antennomere 3 nearly triangle, widened apically, almost as long as wide, in dorsal view partially covering ventral plate of antennomere 4 below; antennomere 4 dorsally (looking from above) almost 1.7 times longer than wide, long oval, made up by a dorsal and a ventral plate. Dorsal plate surface convex and semi-circular in lateral view, lateral sides nearly parallel, anterior side nearly straight; ventral plate completely covered by dorsal plate except apically in dorsal view, surface weakly convex, left lateral sides nearly straight and coincide with those of dorsal plate, right lateral sides distinctly concave and expose part of dorsal plate; anterior side nearly straight. Maxilla with terminal palpomere 2.5 times as long as wide, sides strongly tapering towards apex, arcuate apically.

***Thorax***. Pronotum with anterior margin weakly sinuate; sides subparallel at apical 1/3, from there posteriorly suddenly constricted, and at basal 1/4 subparallel; anterior and lateral edges very narrowly bordered; disc rather convex, median furrow distinct, anterior 3/4 deep and with one longitudinal stria on each side, posterior 1/4 raised into a longitudinal ridge. Pronotal surface polished between punctures, punctation rather dense and strongly fine; lateral sulci distinct, linear, extending to about 1/3 of pronotal length nearly; basal sulcus distinct, sinuate. Prosternal process very narrow, not extending posteriorly beyond front coxae; sides weakly concave, acutely rounded apically. Mesoventrite with intercoxal process trapezoidal in shape, extending beyond 1/2 length of mesocoxae, disc with a median longitudinal ridge; sides converging towards apex, truncate apically. Elytral surface polished between punctures, and with several obscure longitudinal markings, linear or punctiform; punctations rather dense and strongly fine as prosternal those; sides curved, widest near anterior 1/3 of elytral length; then strongly converging apically; Humeri strongly prominent and raised into a slender longitudinal ridge, extending to anterior 1/3 of elytral length; scutellum oval, each sides with a short broad longitudinal ridge, extending to anterior 1/6 of elytral length; suture with one longitudinal stria on each side. Apices rounded. Legs with tibia simple, sides widened apically, metatibia with inner not as female, extending in a tooth-shaped projection apically. Wing as Fig. 4.

***Abdomen*** with ventrite 1 nearly as long as three subsequent ventrites combined; ventrites 2–4 subequal in length; ventrite 5 longer than previous 3 ventrites; ventrite 6 nearly semi-circular shape, anterior margin narrower than posterior margin of ventrite 5, curved apically. Male genital segment (Fig. 5) with sternite provided with single, long, rod-like apophysis; dorsal plate undivided.

***Aedeagus*** (Fig. 6). Median lobe/penis rather long and thick, sclerotized, basal 1/4 curved in lateral view; in dorsal and ventral view sides subparallel, apical 1/4 abruptly converged, and here bears long setae each side, acutely rounded apically. Tegmen fused with penis; tegminal strut strongly long and slender, S-shaped in ventral view.

***Measurements* (in mm)**. BL 4.5-5.0, PL 1.1–1.4, PW 1.5–1.6, EL 3.0–3.4, EW 1.7–2.0, PL/PW 0.7–0.9, EL/EW 1.7–1.8, EL/PL 2.4–2.7, EW/PW 1.1–1.3.

#### Distribution.

Malaysia.

#### Remarks.

Arrow (1920) described *Dadocerus nitidus* based on two specimens but did not explicitly designate one as the “holotype” (Fig. 7). The corresponding author of this paper examined these specimens housed in the Natural History Museum, London (NHMUK), and confirmed that their morphological characters and label data match the original description. Furthermore, in the original description, he suggested that “the two specimens are probably males” without having dissected them. To rectify this error and ensure nomenclatural stability, we hereby designate the following specimens from the original syntype series:

• Lectotype (here designated): Female specimen, collection number 016509885, deposited at the Natural History Museum, London.

• Paralectotype (here designated): Female specimen, labeled “type”, collection number 016509887, deposited at the Natural History Museum, London.

### Genus Pleganophorus

Taxon classificationAnimaliaColeopteraEndomychidae

Hampe, 1855

3C675D31-F1E3-5975-9FB9-04F330418144

Pleganophorus Hampe, 1855: 97.

#### Description (modified from Strohecker 1953).

Body 3.1-3.5 mm long; brown; weakly convex. Dorsal clothed with minute hairs; densely and coarsely punctate. Antenna 4- segmented, shorter than head and thorax together; terminal antennomeres greatly elongated, distinctly longer than the first three segmented combined; reniform in male and allantoiform in female. Pronotum distinctly narrowed, sides gradually widened apically, median groove absent; anterior angles rounded; posterior angles produced as heavy spines, and inner side of each bears a deep fovea basally. Prosternal process narrowly separates front coxae. Elytra strongly elongate, posteriorly tapering, separately rounded at apex; humeri weakly prominent. Tarsal formula 4-4-4 in both sexes.

#### Diagnosis.

The *Pleganophorus* resembles two other genera of Pleganophorinae in appearance. However, the pronotum with the posterior angles developed into long, sharp spines can separate it from *Dadocerus* and *Trochoideus* (Strohecker 1953).

### Pleganophorus bispinosus bispinosus

Taxon classificationAnimaliaColeopteraEndomychidae

Hampe, 1855

4C4A5850-47C0-5BA8-923B-FC78D8E81D2D

Fig. 8

Pleganophorus bispinosus Hampe, 1855: 98.

#### Material examined.

Greece • 1♂; *Pleganophorus bispinosus* Hampe; Morea, Cumani, Brenske.; G. Lewis. 1910-248.; NHMUK 016509888; (NHMUK); GREECE • 1♀; Morea, Cumani, Brenske.; G.Lewis. 1910-248.; NHMUK 016509889; (NHMUK).

**Figure 8.**
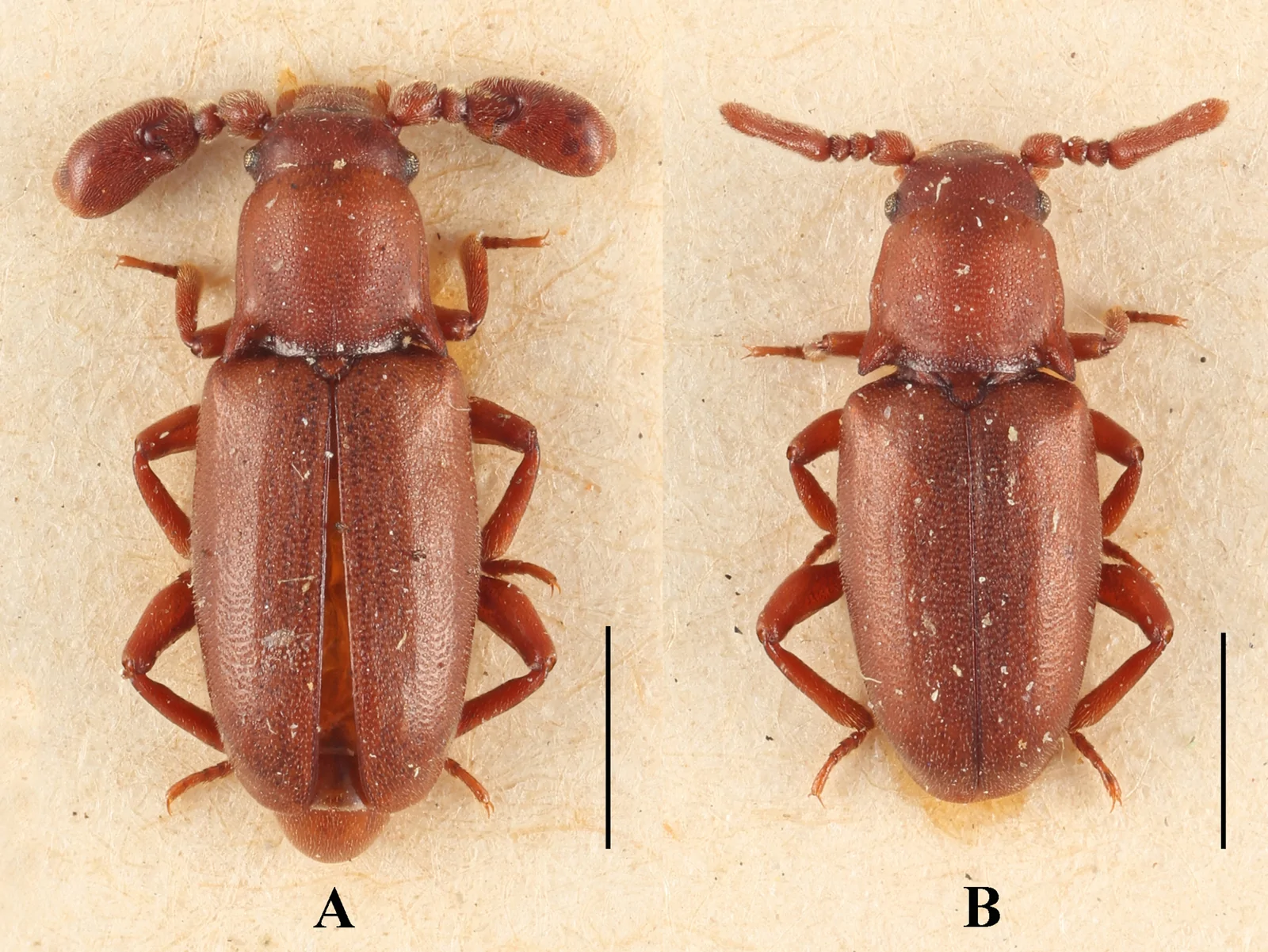
Habitus of *Pleganophorus bispinosus bispinosus*. **A**. Male; **B**. Female. Scale bar: 1.0 mm.

#### Measurements (in mm).

BL 3.1-3.5, EW 1.5–1.6.

#### Distribution.

Bulgaria, Croatia, Czech Republic, Greece, Hungary, Romania and Slovakia (Shockley et al. 2009a).

### Genus Trochoideus

Taxon classificationAnimaliaColeopteraEndomychidae

Westwood, 1833

A80CADAF-58AA-52AB-9058-A02F9DFA01AC

Trochoideus Westwood, 1833: 673. Type species: Paussus cruciatus Dalman, 1825.
Trochoides Chapuis, 1876: 147. Unnecessary replacement name.
Pseudopaussus Schulze, 1916: 292. Type species: Pseudopaussus monstrosus Schulze, 1916.

#### Description (modified from Tomaszewska 2000).

Body 3.0-4.2 mm long, weakly elongate, moderately convex, shiny. Dorsal surfaces with dense and rather short pubescence; densely and confusedly punctate. Colour light to dark brown, sometimes with contrasting markings on the elytra. Antenna with 4 antennomeres (but there are species of *Trochoideus* with females that have 7 antennomeres), almost as long as head and thorax together; terminal antennomeres greatly enlarged, especially in male. Mandible with large tooth and two smaller subapical teeth. Maxilla with palpomere 1 smallest; palpomere 2 twice as long as 1 or 3; terminal palpomere longer than 2 and 3 combined, cylindrical, tapering, narrowly rounded apically. Labium with palpi widely separated at base, terminal palpomere large, subquadrate. Pronotum widest near midlength; lateral edges densely and finely denticulate and setose; basal sulcus hardly visible; lateral sulci in form of shallow, triangular depressions; anterior angles rounded; posterior angles slightly produced, acute. Prosternal process very narrow, extends to posterior margin of coxae, rounded apically. Mesoventral process narrow and elongate, not extending beyond posterior margin of mesocoxae. Elytral epipleuron narrow, incomplete apically. Leg with tibia scarcely widening towards tarsus, surrounded by short, spines apically. Tarsal formula 4-4-4 in both sexes; tarsomeres 1 and 2 weakly lobed ventrally; tarsomeres 3 slightly shorter than tarsomeres 2. Abdomen with six freely articulated ventrites; ventrites 2 and 3 subequal in length. Aedeagus comparatively short, weakly sclerotized, scarcely curved. Tegmen with large tegminal plate; tegminal strut submembranous, slender, slightly shorter than tegminal plate.

#### Diagnosis.

*Trochoideus* resembles two other genera of Pleganophorinae in appearance. However, the pronotum without a median sulcus can separate it from *Dadocerus*, which has the pronotum with a median longitudinal sulcus; and the pronotum with posterior angles not spinose can separate it from *Pleganophorus*, which has the pronotum with the posterior angles developed into long, sharp spines (Strohecker 1953).

##### Species of *Trochoideus* distributed in China

### Trochoideus desjardinsi

Taxon classificationAnimaliaColeopteraEndomychidae

Guérin-Méneville, 1838

F86EFE69-2AEE-52B2-95B0-A4E0DFE7E733

Fig. 9

Trochoideus amphora Cantor, 1846: 282.
Trochoideus termitophilus Roepke, 1919: 34.
Trochoideus rouyeri Pic, 1922: 8.
Trochoideus particularis Pic, 1922: 8.
Pseudopaussus monstrosus Schulze, 1916: 292.

#### Diagnosis.

*Trochoideus desjardinsi* is similar to its congeners from China (*T. sinensis* and *T. tonkineus*). However, *T. desjardinsi* can be separated from both these species by having the dorsal surface of the body densely pubescent; antennomeres 4 in males broadly clavate (2.2 times as long as wide), and nearly parallel-sided, in females narrowly clavate (2.8 times as long as wide) and distinctly converging posteriorly.

**Figure 9.**
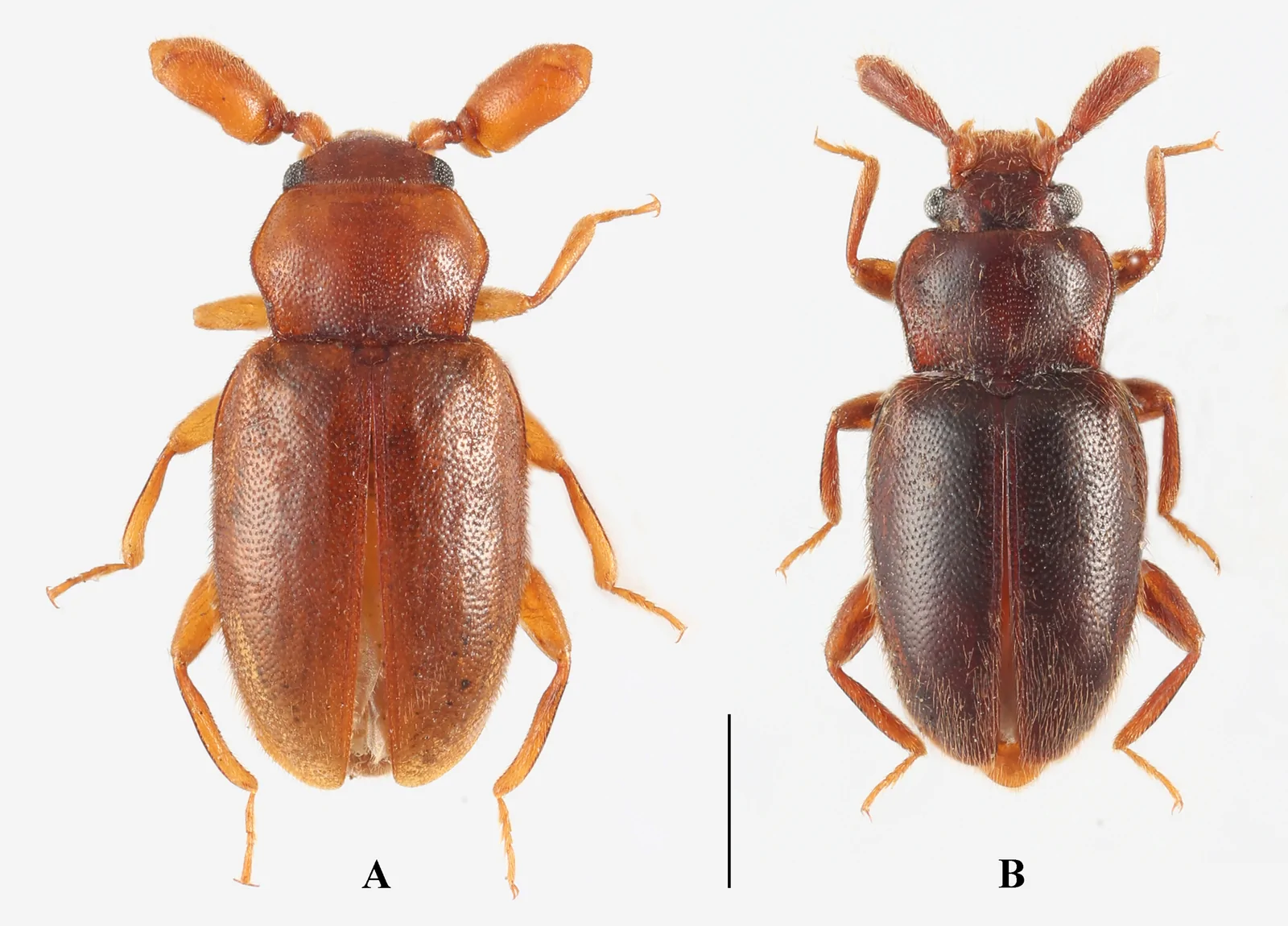
Habitus of *Trochoideus desjardinsi*. **A**. Male; **B**. Female. Scale bar: 1.0 mm.

#### Material examined.

**China** – Hainan • 1♂; Danzhou City (儋州市), Xipei Farm Peilan Team (西培农场培兰队); 2023.VIII.11; (CCLX). **Japan** • 1♂; Is. Chichi-jima, Bonin Islands; 1976.VII.7; Y. Kurosawa.; (NSMT). **Vietnam** • 1♂; Host: wood chip of *Melaleuca* sp.; 2009.III.7; Gang Zhao leg.; IN2950-1-001; (MHBU); 1♀; Host: wood chip; 2009.IV.7; Xiao-Bu Guo leg.; IN2950-1-003; (MHBU); 1♂; ditto except “IN2950-1-004”; (MHBU); 1♂; ditto except “Host: wood chip of *Melaleuca* sp.; Gang Zhao leg.; IN2950-1-005”; (MHBU); 1♀; ditto except “2009.IV.9; Xiao-Bu Guo leg.; IN2950-1-009”; (MHBU); 1♀; Host: wood chip of *Melaleuca* sp.; 2009.III.5; Pu-Ju Yang leg.; IN2950-1-010; (MHBU). BORNEO • 1♂; Sarawak, 1865-66, coll. G. Doria/Mus. Zool. Polonicum Warszawa, 12/45; (MZPW); 1♀; Isole Key, 1873, O. Beccari/Mus. Zool. Polonicum Warszawa, 12/45; (MZPW).

#### Distribution.

Cuba, Fiji, India, Indonesia, Japan, Kenya, Madagascar, Malaysia, Mauritius, Micronesia, Myanmar, Papua New Guinea, Philippines, Samoa, Seychelles, Sri Lanka, Tanzania, Thailand, Uganda, United States of America, Vanuatu. China: Hainan (**new provincial record**) and Taiwan (Shockley et al. 2009a; Berrazueta et al. 2024; Shûhei Nomura et al. 2025).

#### Ecology.

*Trochoideus desjardinsi* has been observed inhabiting the nest of *Anoplolepis gracilipes* (Formicidae) (personal communication). Also, found associated with *Plagiolepis longipes* (Formicidae), *Eutermes ceylonicus*, and *Termes gilvus*. As reported in table 3 of Shockley et al. (2009b), this may indicate a symbiotic relationship between the two taxa.

#### Remark.

“As indicated by the above synonyms, this species was originally spelled ‘dejardinsii’, but this is regarded as an incorrect original spelling under ICZN Article 32. Notably, the spelling ‘Trochoideus Desjardinsi’, which is potentially the correct original spelling *sensu* the Code, appears on p. 334 of the same volume of Revue Zoologique, la Société Cuvierienne (1838), in the ‘Table alphabétique’. Subsequent authors have adopted this spelling, whereas the original spelling has not been used. Therefore, because ‘desjardinsi’ qualifies as the prevailing usage (ICZN Article 33.3.1), this spelling should be maintained” (Shûhei Nomura et al. 2025).

### Trochoideus sinensis

Taxon classificationAnimaliaColeopteraEndomychidae

Li & Chang sp. nov.

2DA4CD27-C80B-5876-A058-56DC9A790212

https://zoobank.org/6D57A64D-4E72-4732-8E1E-A3E84EC411FE

Figs 10, 11, 12, 13, 14, 15, 16, 17

#### Type material.

***Holotype***. China – **Guangdong Prov**. • ♂; Guangdong, Shaoguan City (韶关市), Shixin County (始新县) Chebaling (车八岭), 24.75736°N, 114.198523°E; 714 m; 2023.VI.18; Pan-Pan Li leg.; (IZCAS); ***Paratypes***. China • 48♂♂45♀♀; same collecting data as holotype; (IZCAS); China – **Guangdong Prov**. • 2♂♂3♀♀; ditto except (NHMC); China – **Guangdong Prov**. • 2♂♂; ditto except dissected (NHMC); China – **Guangdong Prov**. • 6♂♂5♀♀; ditto except “24.711691°N, 114.24619°E; 445 m; 2022.VIII.31”; (IZCAS); China – **Guangdong Prov**. • 2♂♂; ditto except (NHMC); China – **Guangdong Prov**. • 2♂♂2♀♀; Guangdong, Shaoguan City, Shixin County, Chebaling; 2008.VII.22; Xiao-Yu Zhu leg.; (CBWX); China – **Jiangxi Prov**. • 2♂♂; Jiangxi, Longnan City (龙南市), Jiulianshan (九连山), Xiagongtang (虾公塘), 24.580078°N, 114.437737°E; 717 m; 2020.IX.15; Ming-Min Ma leg.; (NHMC).

**Figure 10.**
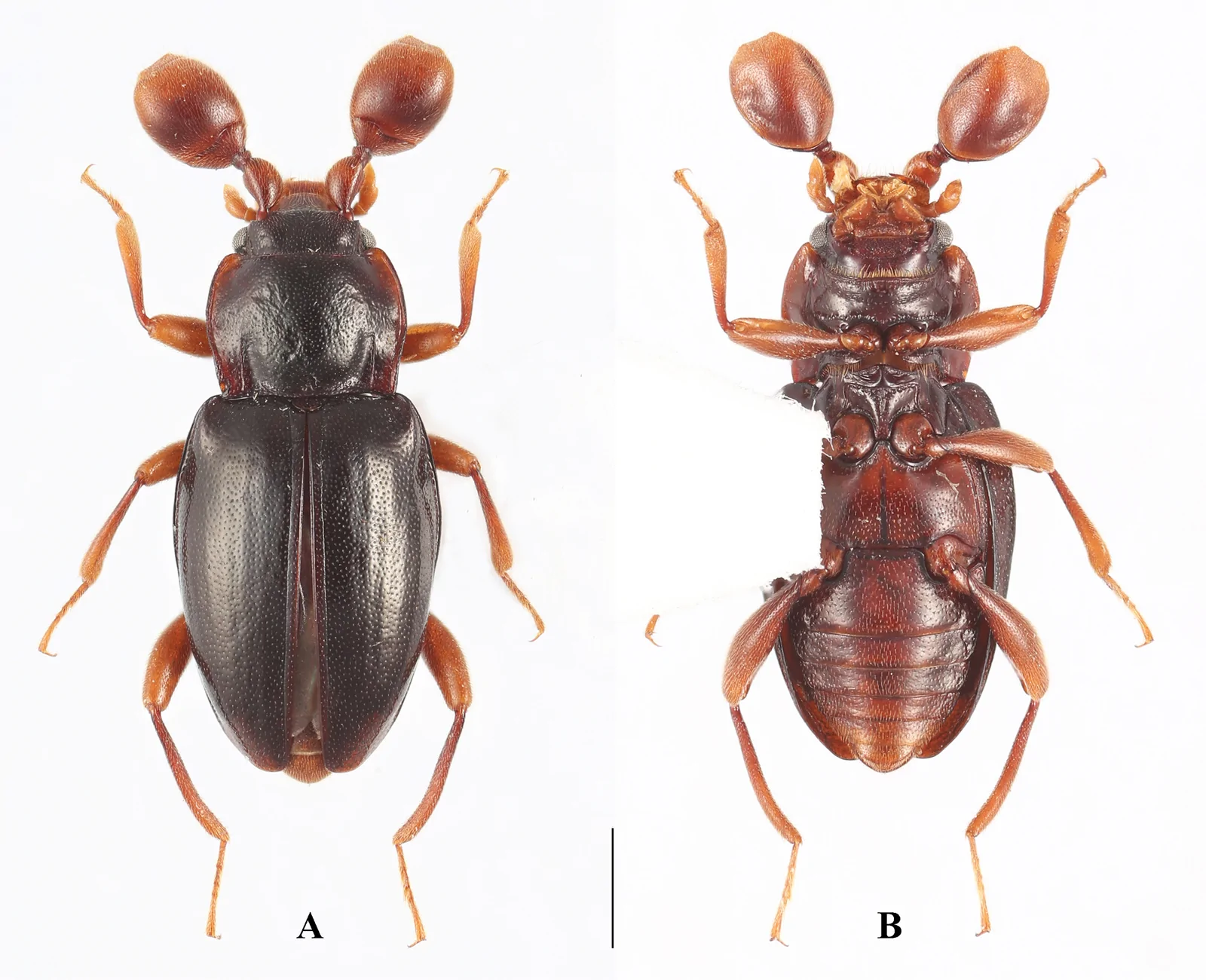
Habitus of *Trochoideus sinensis* sp. nov. (male). **A**. Dorsal view; **B**. Ventral view. Scale bar: 1.0 mm.

**Figure 11.**
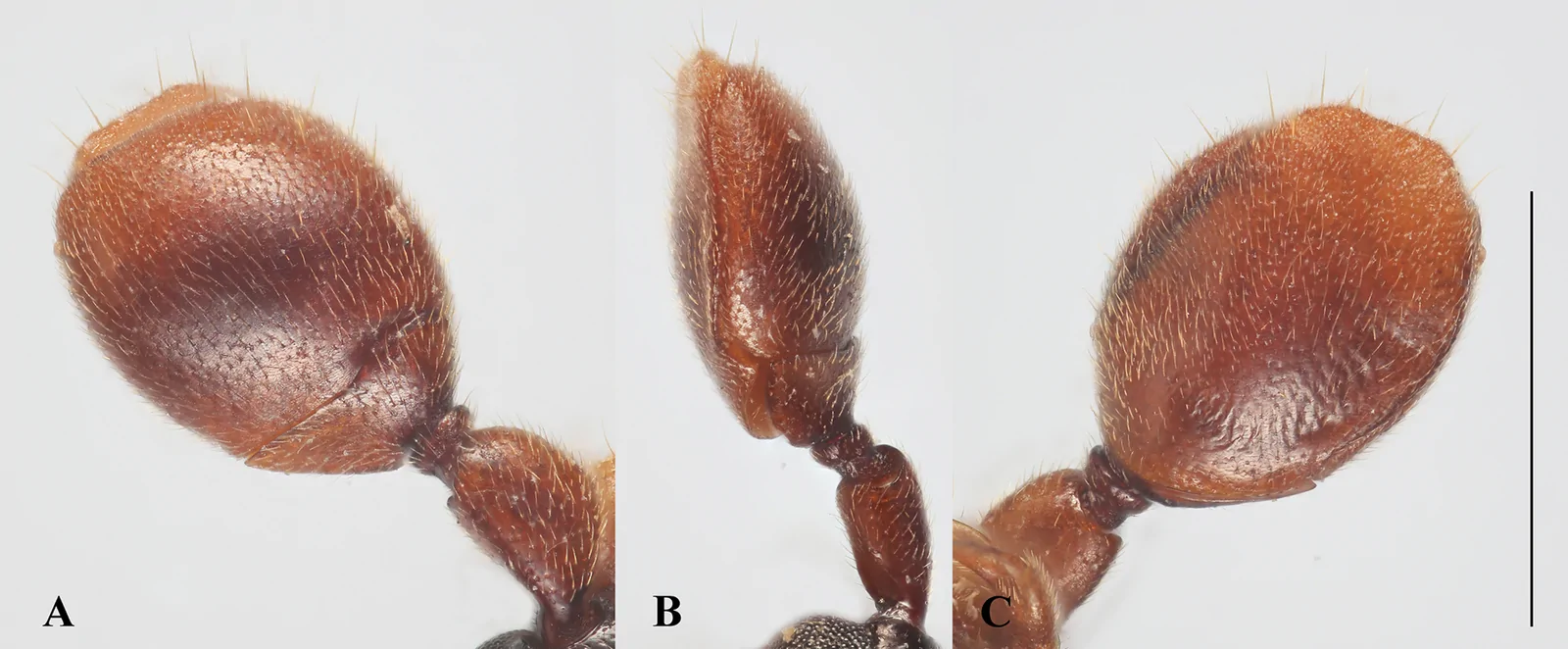
Left antenna of *Trochoideus sinensis* sp. nov. (male). **A**. Dorsal view; **B**. Lateral view; **C**. Ventral view. Scale bar: 1.0 mm.

**Figure 12.**
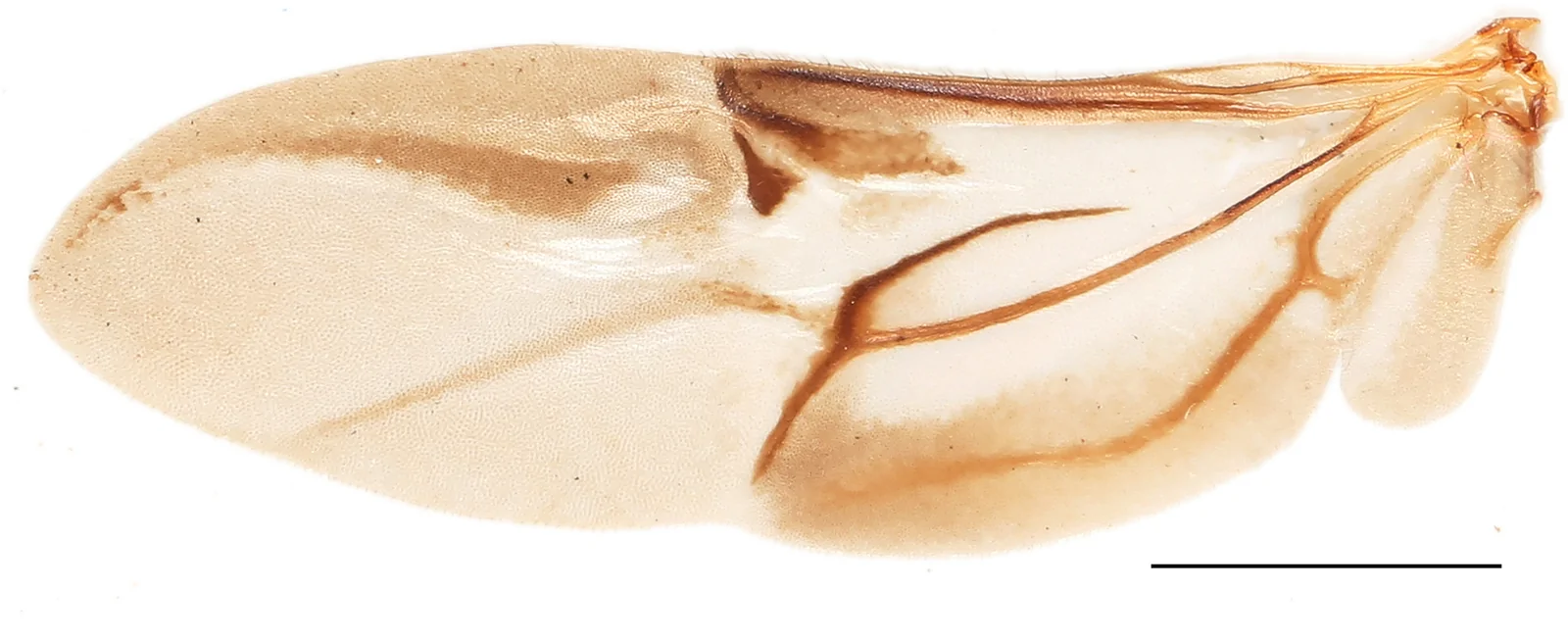
Left hind wing of *Trochoideus sinensis* sp. nov. Scale bar: 1.0 mm.

**Figure 13.**
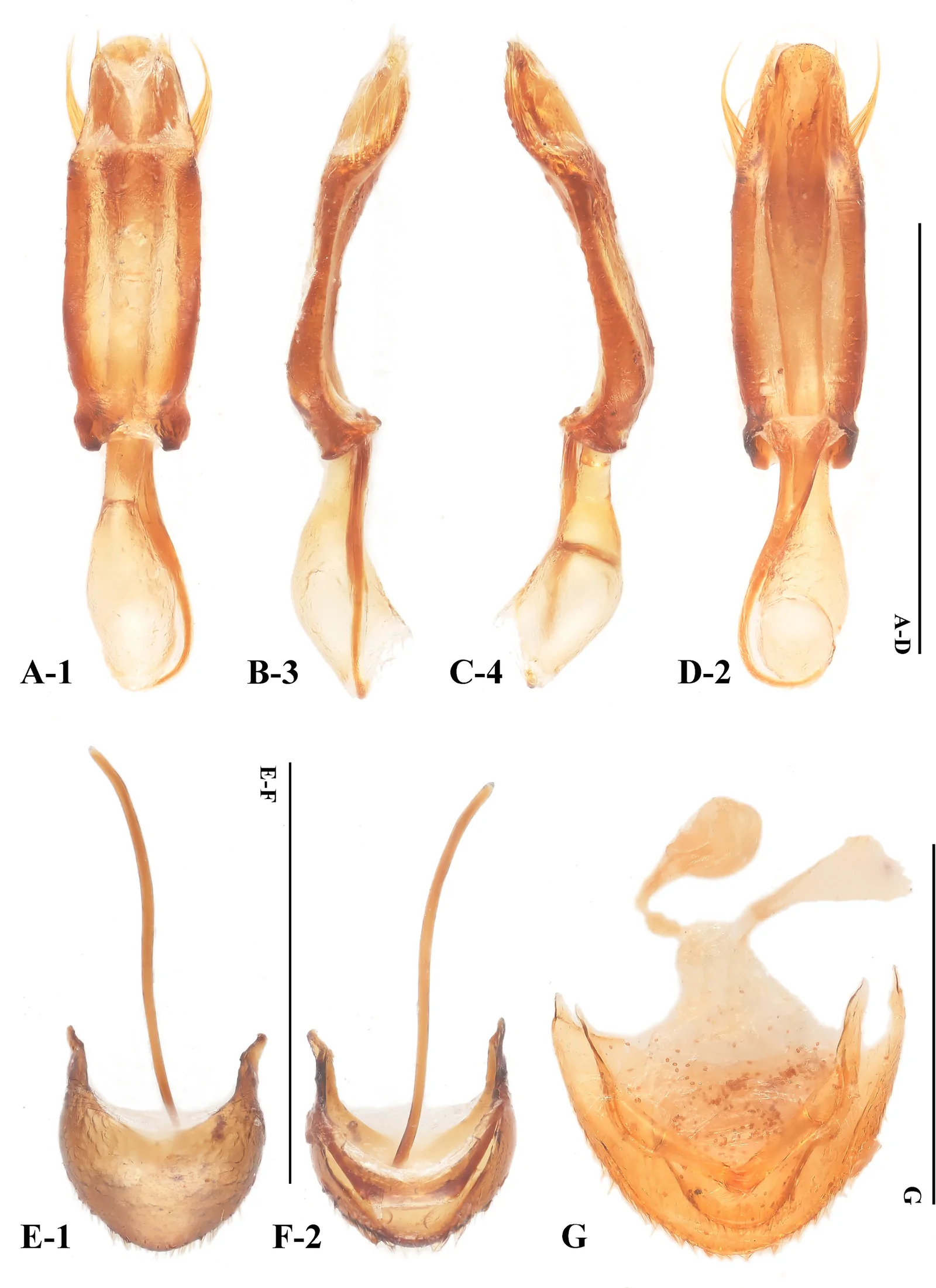
Genitalia and male genital segment of *Trochoideus sinensis* sp. nov. **A–D**. Aedeagus; **E, F**. Male genital segment; **G**. Female genitalia; 1 dorsal view 2 ventral view 3 left lateral view 4 right lateral view. Scale bar: 1.0 mm.

**Figure 14.**
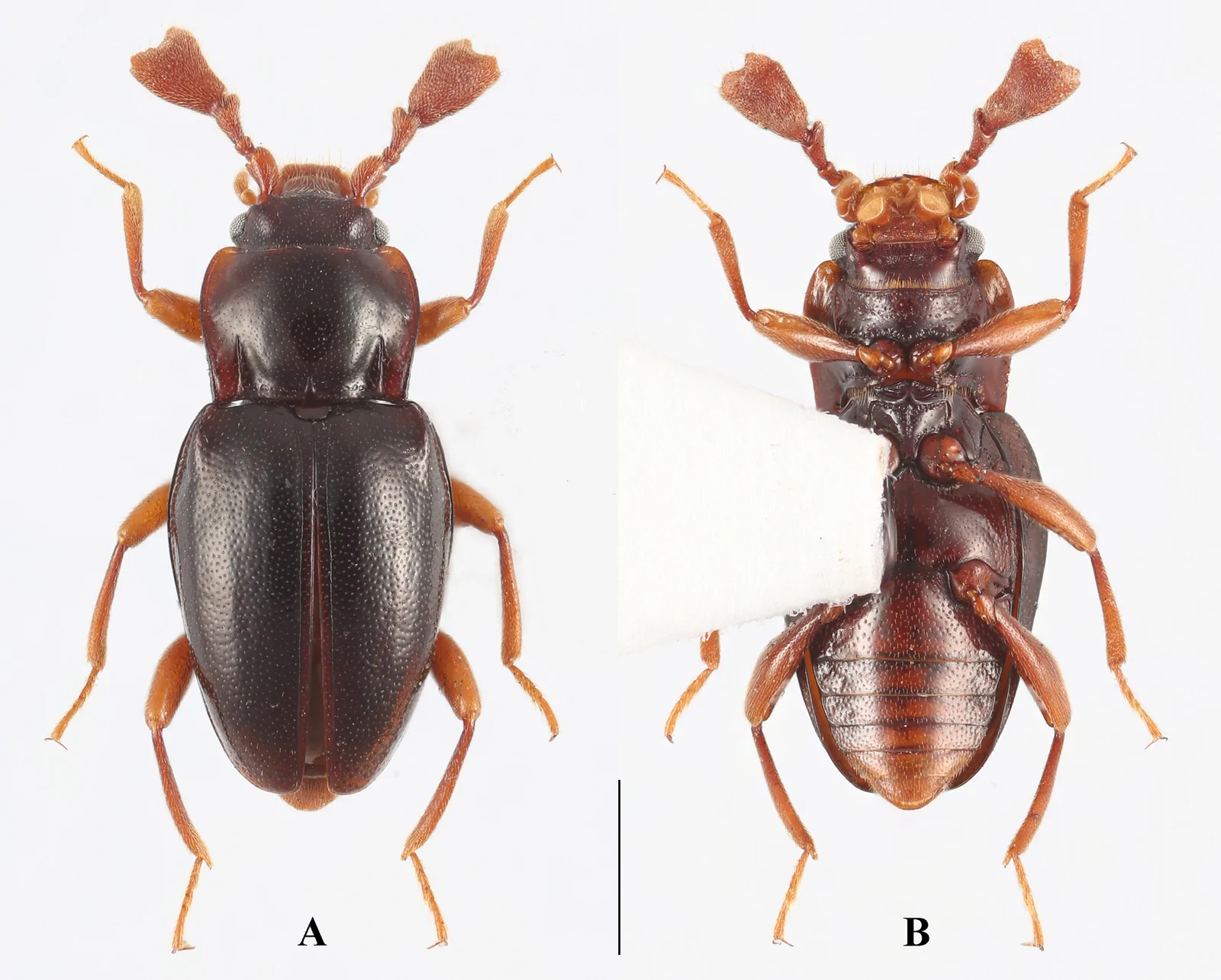
Habitus of *Trochoideus sinensis* sp. nov. (female). **A**. Dorsal view; **B**. Ventral view. Scale bar: 1.0 mm.

**Figure 15.**
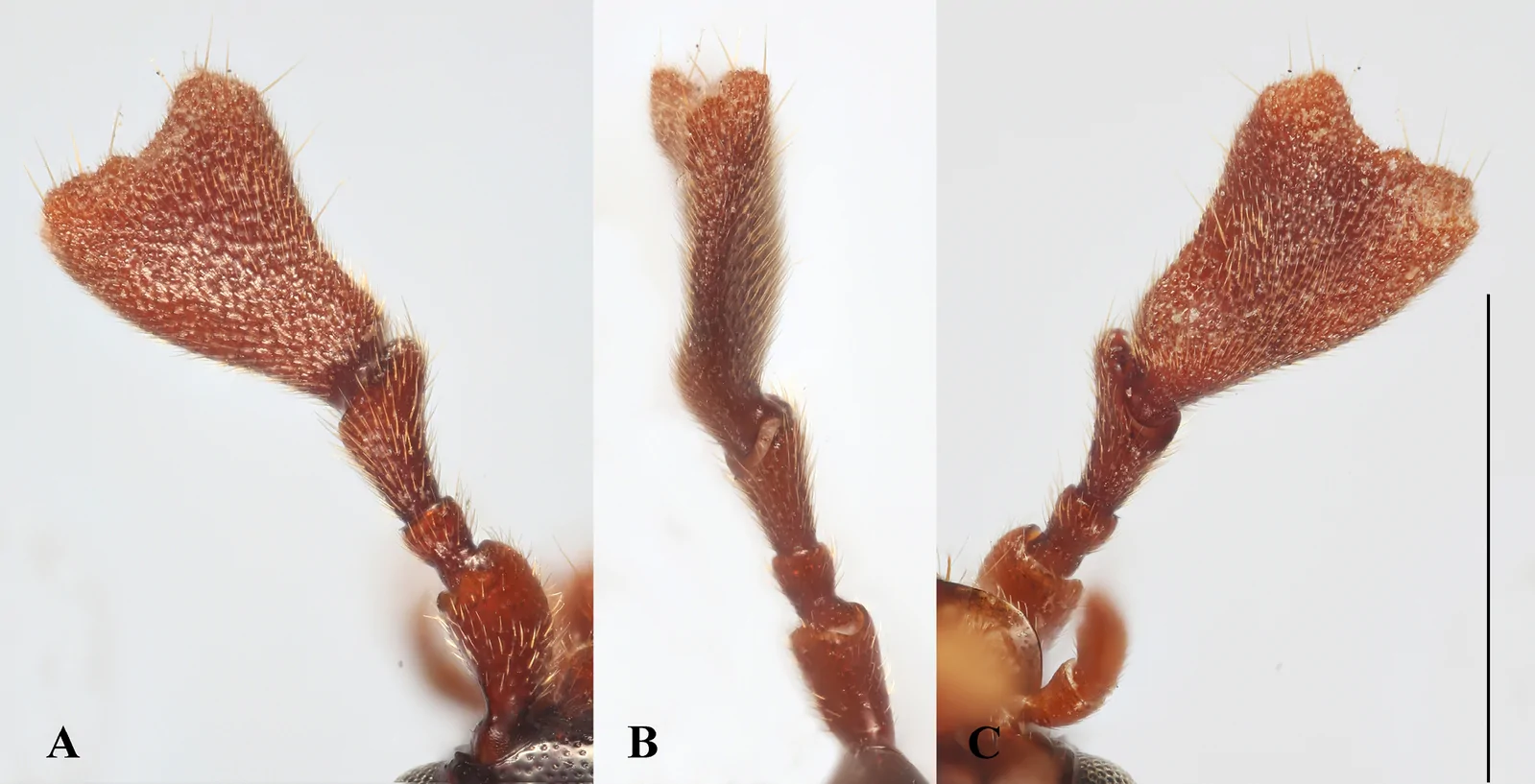
Left antenna of *Trochoideus sinensis* sp. nov. (female). **A**. Dorsal view; **B**. Lateral view; **C**. Ventral view. Scale bar: 1.0 mm.

**Figure 16.**
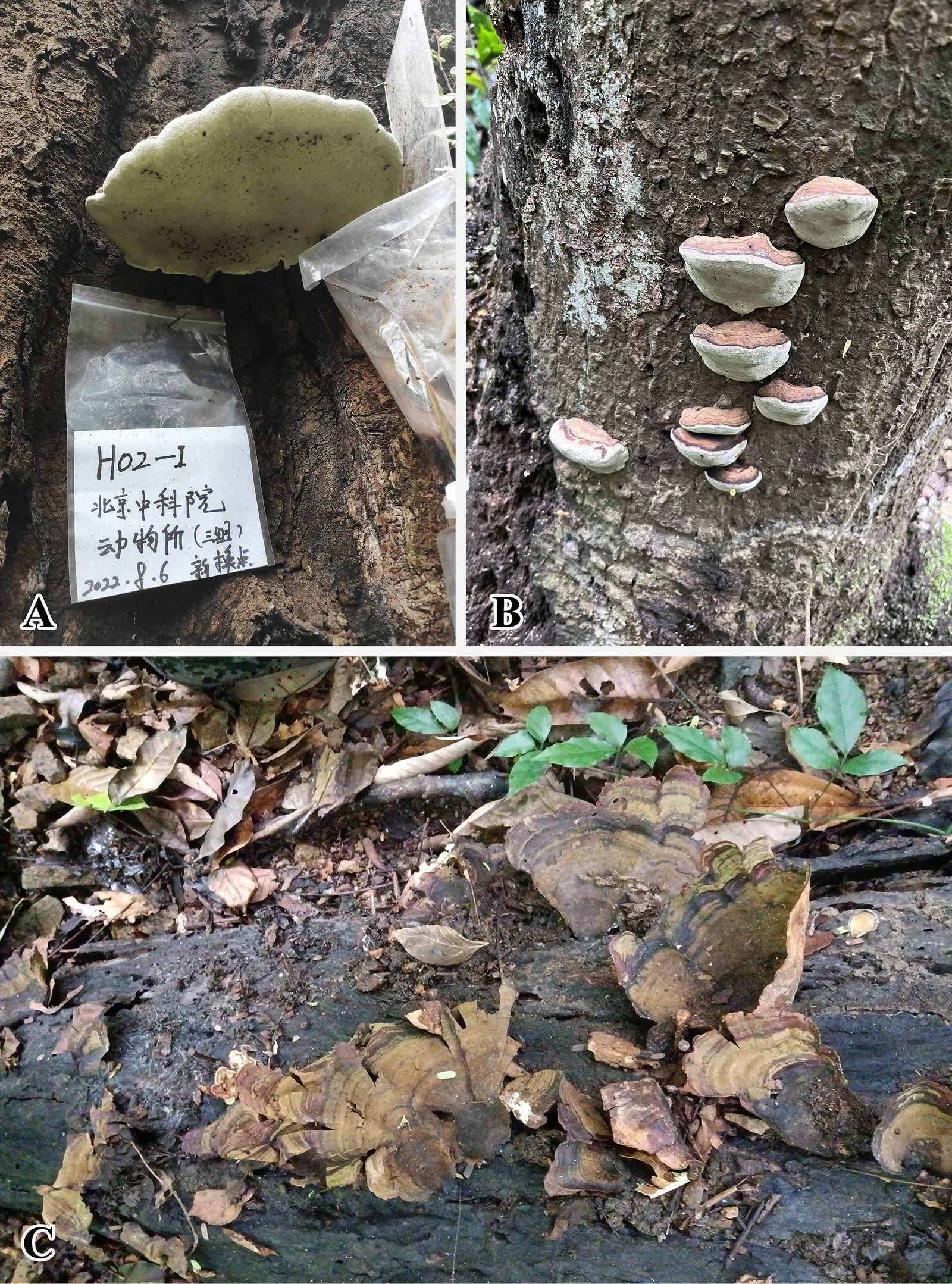
Tree-inhabiting fungi attract *Trochoideus sinensis* sp. nov. **A**. *Sanghuangporus* sp.; **B**. *Ganoderma applanatum*; **C**. *Stereum ostrea*.

**Figure 17.**
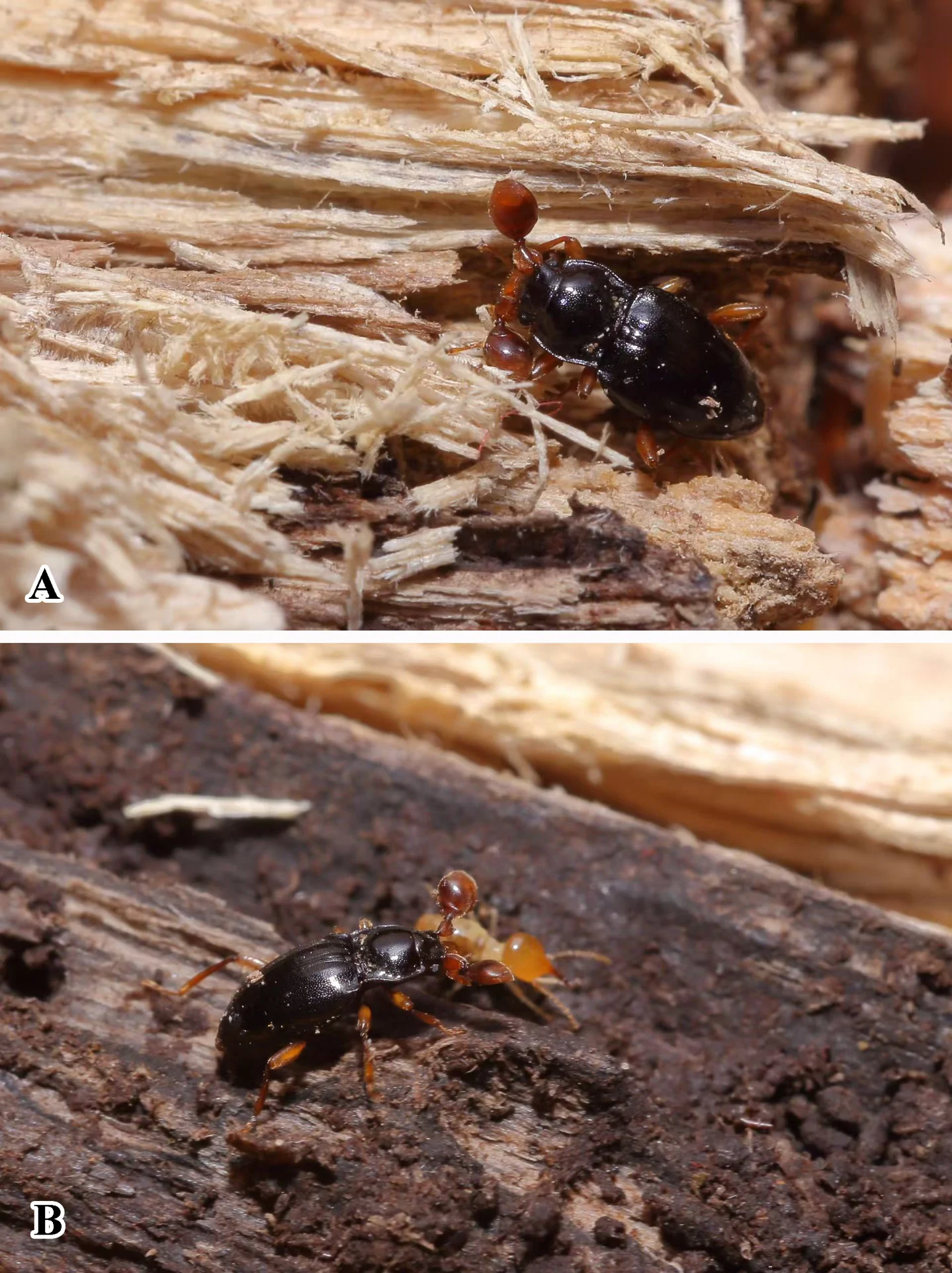
Habitats and habitus of *Trochoideus sinensis* sp. nov. **A**. Adults of *Trochoideus sinensis* sp. nov. inhabit the subcortical space of decaying trees; **B**. Adults of *Trochoideus sinensis* sp. nov. exhibit a possible symbiotic association with *Nasutitermes* sp. (Photos by Zhen-Hua Liu).

#### Diagnosis.

*Trochoideus sinensis* sp. nov. is similar to *T. tonkineus* in having a dark brown, glabrous and elongate-oval body. However, *T. sinensis* sp. nov. can be separated from *T. tonkineus* by the antennomere 4 in male broadly oval, 1.4 times as long as wide (vs. elongate oval, 2.0 times as long as wide, see Fig. 19), and in female in shape of elongated triangle, widely emarginate apically (vs. sides near parallel, tridentate apically, see Fig. 20); surfaces of the pronotum and elytra with coarse punctures (vs. fine punctae); and the pronotum with anterior margin weakly arcuate medially (vs. concave medially).

#### Description.

**Male. *Body*** (Figs 10, 11, 12, 13, 16) length 4.9–5.2 mm; Body long and oval, 2.2–2.5 times as long as wide; weakly convex; smooth, rather shiny. Dorsal surfaces mostly dark brown with antenna, clypeus, labrum, mandible and leg light brown; ventral surfaces dark brown but lighter than dorsal surfaces, elytral epipleura of the same colour as dorsal surfaces.

***Head***. Antenna (Fig. 11) 4 segmented, scape large, with inner margin arcuate, about 1.6 times longer than wide; pedicel slightly wider than long, base partially obscured covered by scape, widest apically; in dorsal view antennomere 3 strongly transverse, widened apically, about 2.0 times as wide as long, in dorsal view covering most of ventral plate of antennomere 4 below it; antennomere 4 dorsally (looking from above) almost twice as long as wide, wide oval, made up by a dorsal and a ventral plate. Dorsal plate surface convex and semi-circular in lateral view, lateral sides curved, anterior side nearly straight; ventral plate completely covered by dorsal plate except apically in dorsal view, surface weakly convex, lateral sides curved and coincide with those of dorsal plate, anterior side wide with shallow concavity apically. Maxilla with terminal palpomere 1.5 times as long as wide, sides strongly tapering towards apex, arcuate apically.

***Thorax***. Pronotum 1.2–1.4 mm long, 1.5–1.8 mm wide. Anterior margin nearly straight; sides curved, widest near 1/2 of pronotal length; anterior and lateral edges very narrowly bordered; disc rather convex, median furrow absent; pronotal surface polished between punctures, punctation rather dense and strongly coarse; lateral sulci distinct, linear, extending to about 1/3 of pronotal length; basal sulcus indistinct. Prosternal process very narrow, not extending posteriorly beyond front coxae; sides nearly straight, acutely rounded apically. Mesoventrite with intercoxal process trapezoidal in shape, extending beyond 1/2 length of mesocoxae, sides converging towards apex, truncate apically. Elytra 3.3–3.7 mm long, 2.0–2.4 mm wide; elytral surface polished between punctures, punctations coarser and denser than those on pronotum; sides curved, widest near anterior 1/4 of elytral length; then converging apically; humeri weakly prominent; apices rounded. Leg with tibia simple, distinctly widened apically, metatibia with inner sides weakly sinuate. Wing as Fig. 12.

***Abdomen*** with six freely articulated ventrites. Ventrite 1 nearly as long as three subsequent ventrites combined; ventrites 2–4 subequal in length; ventrite 5 longer than previous 3 ventrites; ventrite 6 nearly semi-circular in shape, anterior margin narrower than posterior margin of ventrite 5, weakly curved apically. Male genital segment (Fig. 13E, F) with sternite provided with single, long, rod-like apophysis; dorsal plate undivided.

***Aedeagus*** (Fig. 13A–D). Median lobe/penis rather long and robust, sclerotized, basal 1/6 curved in lateral view; sides subparallel, apical 1/4 abruptly converged, broadly rounded apically in dorsal and ventral views. Tegmen fused with penis; parameres bear long setae apically; tegminal strut long and slender, distinctly curved apically.

**Female** (Figs 14, 15). Length 4.7–4.8 mm. Antenna (Fig. 15) with pedicel weakly longer than wide and more elongate than pedicel of male; antennomere 3 strongly elongate, widened apically; antennomere 4 elongated, triangular, deeply and widely emarginate apically; left lateral side curved, right lateral side emarginate; ventral surfaces of antennomere 4 with disc concavity. Tibiae more slender than in male, with sides weakly widened apically; metatibia with inner edge extending in a tooth-shaped projection apically. Abdominal ventrite 6 with posterior margin rounded apically. Female genitalia as Fig. 7G; coxites separated without styli, densely setose apically; spermatheca small, oval; accessory gland elongate oval; sperm duct short.

***Measurements* (in mm)**. BL 4.7–5.2, PL 1.2–1.4, PW 1.5–1.8, EL 3.3–3.7, EW 2.0–2.4, PL/PW 0.8, EL/EW 1.5–1.7, EL/PL 2.6–2.8, EW/PW 1.3.

#### Etymology.

The specific epithet derives from the Latin root “sin-” referring to the current distribution of this species being confined to China.

#### Distribution.

China: Guangdong and Jiangxi.

#### Ecology.

Specimens of *Trochoideus sinensis* sp. nov. from Guangdong were collected using a Mycophagous Insect Window Trap on a tree bearing fungi. Based on Internal Transcribed Spacer (ITS) sequencing, the associated fungi were identified as *Ganoderma applanatum*, *Sanghuangporus* sp. and *Stereum ostrea* (Fig. 16).

The observed co-occurrence of *Trochoideus sinensis* sp. nov. with the termite *Nasutitermes* sp. (Termitidae) beneath the bark of a decaying tree suggests a potential symbiotic association between these taxa (Fig. 17).

### Trochoideus tonkineus

Taxon classificationAnimaliaColeopteraEndomychidae

Strohecker, 1980

B29322DC-B8B7-55F6-B10E-966DFFEF3006

Figs 18, 19, 20

Trochoideus tonkineus Strohecker, 1980: 92.

#### Material examined.

China – **Hainan** • 1♂1♀; Wuzhishan (五指山); 2014.V.21; (NHMC).

**Figure 18.**
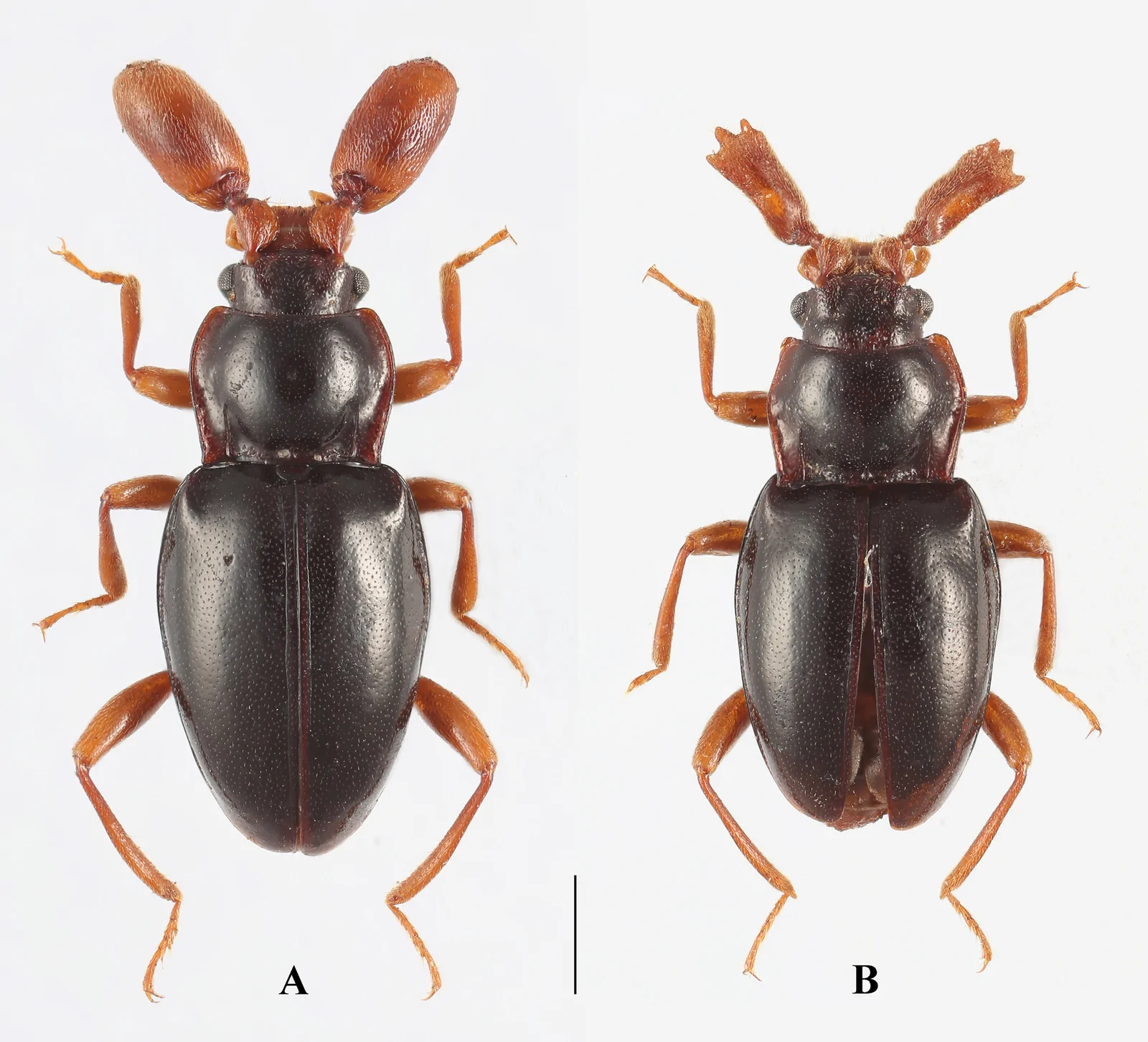
Habitus of *Trochoideus tonkineus*. **A**. Male; **B**. Female. Scale bar: 1.0 mm.

**Figure 19.**
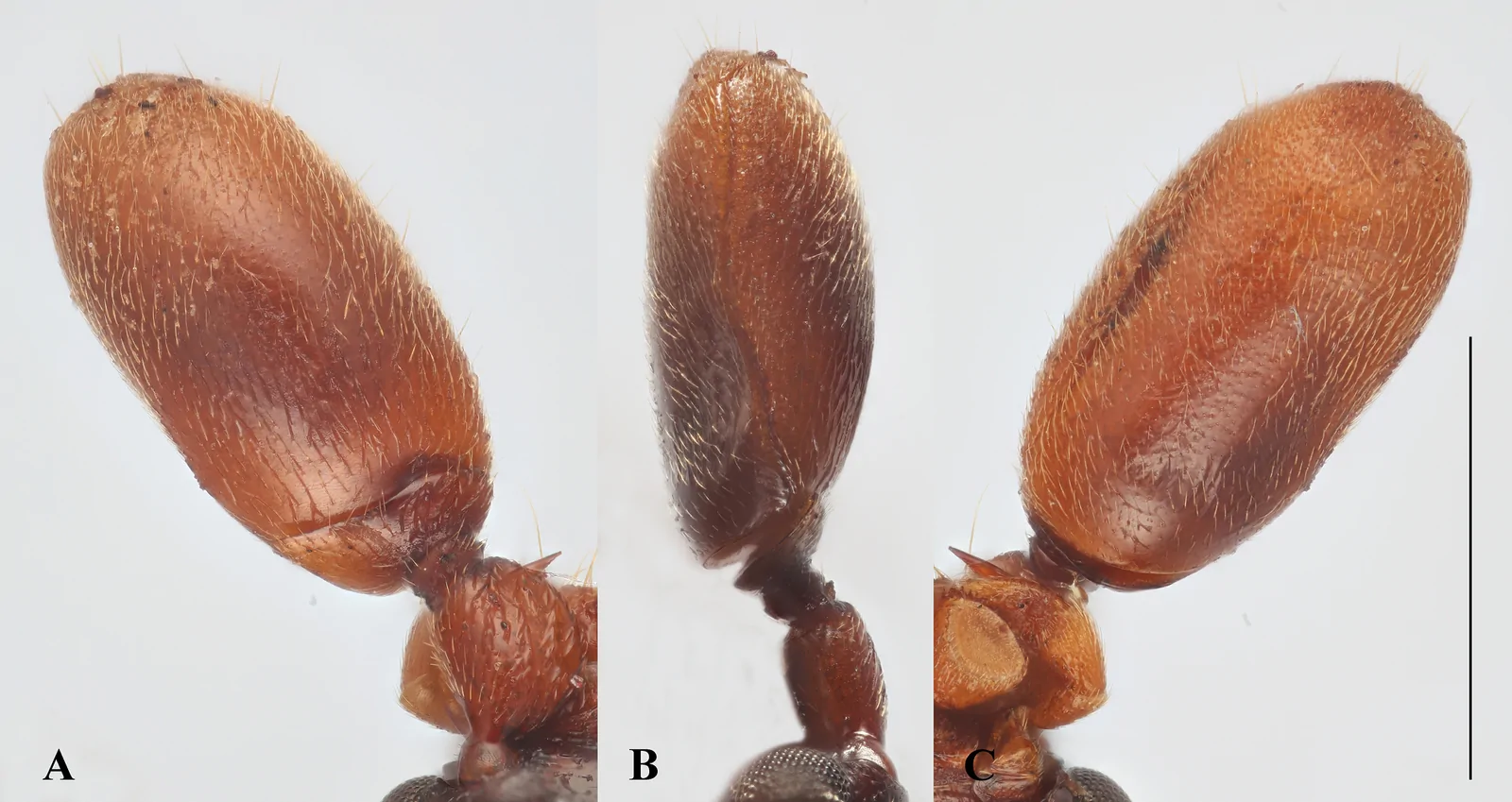
Left antenna of *Trochoideus tonkineus* (male). **A**. Dorsal view; **B**. Lateral view; **C**. Ventral view. Scale bar: 1.0 mm.

**Figure 20.**
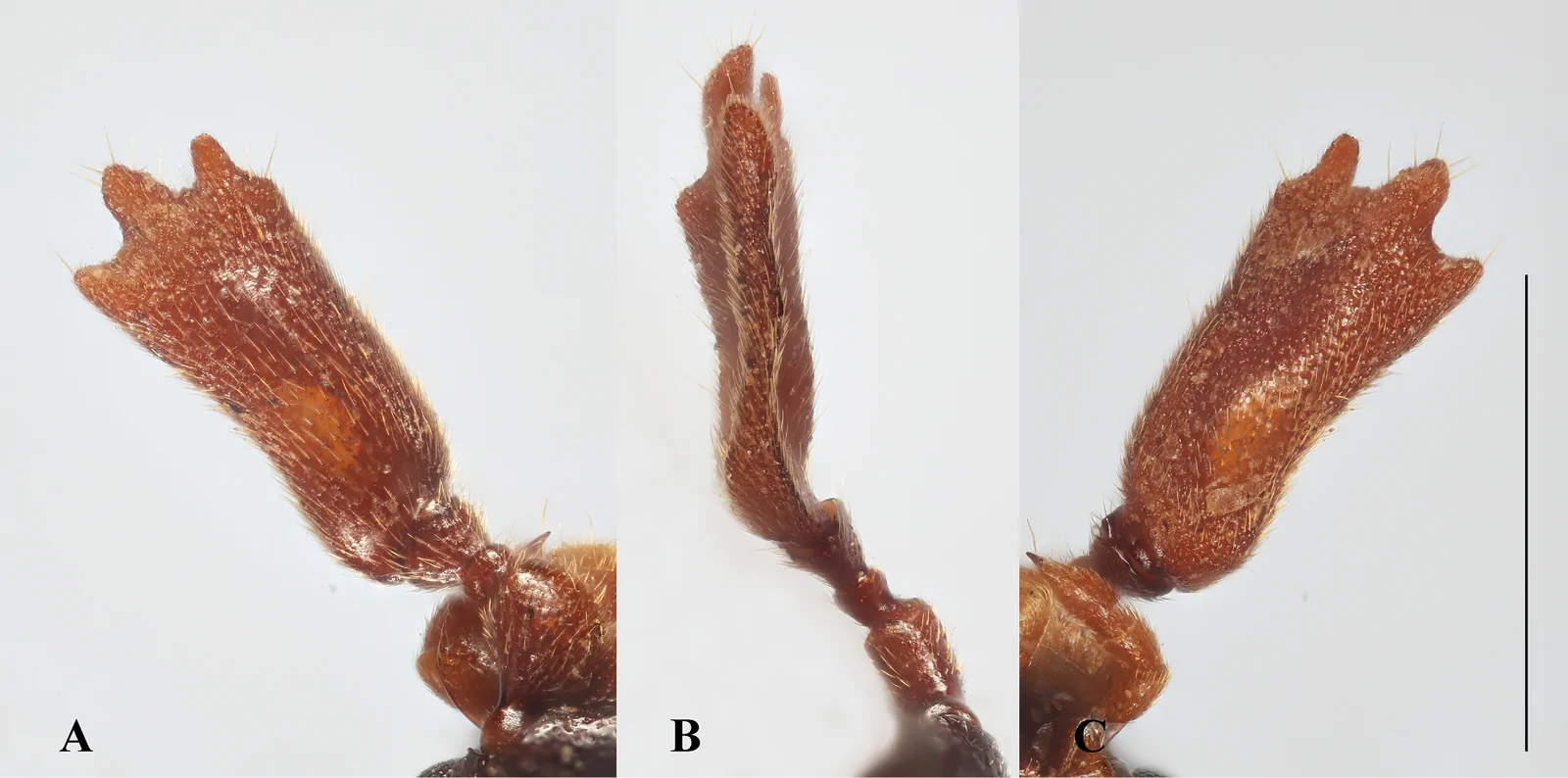
Left antenna of *Trochoideus tonkineus* (female). **A**. Dorsal view; **B**. Lateral view; **C**. Ventral view. Scale bar: 1.0 mm.

#### Diagnosis.

*Trochoideus tonkineus* appears to be similar to *T. sinensis* sp. nov. in having a glabrous, dark brown and elongate-oval body. However, *T. tonkineus* differs from *T. sinensis* sp. nov. by the antennomere 4 in males (Fig. 19) elongate-oval in shape (vs. broadly oval); in females (Fig. 20) sides almost parallel, tridentate apically (vs. elongated triangle, deeply and widely emarginate apically); pronotum and elytra with fine punctures (vs. coarse punctures); and pronotum with anterior margin concave medially (vs. weakly arcuate medially).

#### Description of female.

***Body*** (Figs 18B, 20) length 4.8 mm; body long and oval, 2.0 times as long as wide; weakly convex; smooth, rather shiny. Dorsal surfaces mostly dark brown with antennae, clypeus, labrum, mandibles and legs light brown; ventral surfaces dark brown but slightly lighter than dorsal surfaces, elytral epipleura same colour as dorsal surfaces.

***Head***. Antenna (Fig. 19) 4-segmented, scape subglobose, 1.5 times longer than wide; pedicel longer than wide, concave medially; antennomere 3 nearly triangular, widened apically, 1.8 times wider than pedicel; antennomere 4 nearly parallel-sided, about 2.4 times as long as wide, dorsal and ventral surfaces with deep, wide concavity medially, tridentate apically. Maxilla with terminal palpomere 1.5 times as long as wide, sides strongly tapering towards apex, arcuate apically.

***Thorax***. Pronotum 1.2 mm long, 1.6 mm wide; about 0.8 times as long as wide; anterior margin distinctly concave medially; sides curved, widest near 1/2 of pronotal length; anterior and lateral edges very narrowly bordered; disc rather convex, median furrow absent; pronotal surface polished between punctures, punctation rather dense and fine; lateral sulci distinct, linear, extending nearly to 1/4 of pronotal length, with trianglar concave area medio-basally; basal sulcus poorly developed. Prosternal process distinctly narrower than intercoxal process of mesoventrite, not extending beyond front coxae; sides nearly straight, weakly truncate apically. Mesoventrite with intercoxal process extending to approximate 1/2 length of mesocoxae, nearly parallel-sided, weakly rounded apically. Elytra 3.0 mm long, 2.4 mm wide; 1.3 times as long as wide; 2.5 times longer than pronotum, 1.5 times wider than pronotum; elytral surface polished between punctures, punctations dense, coarser than those on pronotum; sides curved, widest near anterior 1/4 of elytral length; then converging apically; humeri weakly prominent; elytral apices rounded. Legs with fore and mid tibiae simple, gradually widened apically, metatibia with inner edge extending in a triangular tooth at apex.

***Abdomen*** with six freely articulated ventrites. Ventrite 1 nearly as long as three subsequent ventrites combined; ventrites 2–4 subequal in length; ventrite 5 longer than previous 3 ventrites; ventrite 6 nearly semi-circular in shape, rounded apically.

***Measurements* (in mm)**. BL 4.8, PL 1.2, PW 1.6, EL 2.7, EW 2.3, PL/PW 0.8, EL/EW 1.2, EL/PL 2.3, EW/PW 1.4.

#### Distribution.

Vietnam: Tonkin. China (**new country record**): Hainan.

##### Key to the species of *Trochoideus* known in China

**Table:** 

1	Body with dorsal surfaces densely pubescent	***T. desjardinsi***
–	Body with dorsal surfaces glabrous	**2**
2	Antennomeres 4 in male broadly oval (1.4 times as long as wide); antennomeres 4 in female in form of elongated triangle, deeply and widely emarginate apically	***T. sinensis* sp. nov**.
–	Antennomeres 4 in male elongate-oval (2.0 times as long as wide); antennomeres 4 in female subparallel-sided, tridentate apically	***T. tonkineus***

## Supplementary Material

XML Treatment for Pleganophorinae

XML Treatment for Dadocerus

XML Treatment for Dadocerus nitidus

XML Treatment for Pleganophorus

XML Treatment for Pleganophorus bispinosus bispinosus

XML Treatment for Trochoideus

XML Treatment for Trochoideus desjardinsi

XML Treatment for Trochoideus sinensis

XML Treatment for Trochoideus tonkineus

## Additional information

### Artificial Intelligence (AI) use

The authors accept full responsibility for the content of the manuscript, including the disclosure of any use of AI.

No AI tools were used in the preparation of this manuscript.

### Author ORCIDs

M. Bai https://orcid.org/0000-0001-9197-5900

L.-X. Chang https://orcid.org/0009-0003-6174-1195

### Data availability

All of the data that support the findings of this study are available in the main text.
